# Oxytetracycline have the therapeutic efficiency in CD133^+^ HCC population through suppression CD133 expression by decreasing of protein stability of CD133

**DOI:** 10.1038/s41598-018-34301-1

**Published:** 2018-10-31

**Authors:** Yeonhwa Song, In-Ki Kim, Inhee Choi, Se-Hyuk Kim, Haeng Ran Seo

**Affiliations:** 1 Cancer Biology Laboratory, Institut Pasteur Korea, 16, Daewangpangyo-ro 712 beon-gil, Bundang-gu, Seongnam-si, Gyeonggi-do, 13488 Korea; 2 Department of Convergence Medicine, University of Ulsan College of Medicine and Asan Institute for Life Sciences, ASAN Medical center, Olympic-ro 43-gil, Songpa-gu, Seoul, 05505 Korea; 3 Medicinal Chemistry, Institut Pasteur Korea, 16, Daewangpangyo-ro 712 beon-gil, Bundang-gu, Seongnam-si, Gyeonggi-do, 13488 Korea

## Abstract

Cancer stem cells (CSCs) are considered a serious sub-population in cancer tissues because of their strong resistance to conventional chemotherapy and radiotherapy. Thus, the current advancements in the use of liver cancer stem cells (LCSC) to develop efficient and organized means to an antitumor agent is quickly gaining recognition as a novel goal. Previously, we characterized CSCs in primary hepatocellular carcinoma (HCC) and identified CD133 as a CSC cell-surface marker. In this study, we proposed to use non-target based high throughput screening (HTS) approach to specifically target AFP^+^/CD133^+^ HCC present in mixed populations of HCC cells with hepatocytes. Through screening, we identified oxytetracycline, which showed significant inhibition activity of LCSC population without damage on hepatocytes. To determine whether oxytetracycline targets LCSC, we examined whether oxytetracycline treatment could change the CD133 expression, spheroid forming ability as well as the levels of stem cell-related markers. Treatment of spheroid-forming LCSC with oxytetracycline effectively decreased the spheroid formation and the CD133^+^ cell population. oxytetracycline could suppress expression of CD133 without changing of expression of other stem cell-related markers. Importantly, these series of phenomena by oxytetracycline occurs because of alteration of CD133 protein stability by oxytetracycline. Alterations in the malignant properties of AFP^+^/CD133^+^ HCC by oxytetracycline were also investigated by xenograft assay in nude mice. Treatment of oxytetracycline significantly attenuated tumor formation and CD133^+^ cell population in xenograft mice. These results indicate that the oxytetracycline suppresses stemness and malignancies in HCC cells through destabilization of CD133 in LCSC population, providing novel therapeutic strategies targeting specifically cancer stem-like cells.

## Introduction

Hepatocellular carcinoma (HCC) is the seventh common malignant cancer with lung cancer and the third leading cause of cancer-related deaths in the world^1–3^. Most HCCs harbor resistant to conventional chemotherapy. Moreover, patients with HCC usually have poor tolerance of systemic chemotherapy owing to underlying liver dysfunction. The cumulative 3-year recurrence rate after resection is approximately 80%, and recurrence after resection usually results in a high rate of mortality^4^.

Accordingly, liver cancer stem cells (LCSCs) is quickly gaining recognition as a novel goal to develop efficient antitumor agents^5,6^. However, the molecular mechanisms and signaling cascades involved in LCSC innate resistance to radio- and chemotherapy remains elusive, and accordingly, research in these areas will directly translate into acquisition of novel technologies and improved knowledge of fundamental biological knowledge. For this reason, we focused on elucidation of chemotherapy resistance mechanisms in LCSC to define novel target for liver cancer therapy.

In our previous study, we characterized CSCs in primary HCC and identified CD133 as a LCSC cell-surface marker^7^. CD133 (Prominin 1) is a 5-transmembrane glycoprotein expressed by a subpopulation of the hematopoietic stem cells originating from fetal liver and bone marrow^8,9^. CD133 has been considered a surface marker of CSCs in cancers of the brain, colon, pancreas, prostate, and liver. Liver cancer patient with high expression of CD133 displayed short overall survival and high recurrence rates relative to patient with low expression of CD133^10,11^. CD133^+^ cells have resistance to conventional chemotherapy and radiation treatment in HCC. To confer chemo-resistance, CD133^+^ liver CSCs can modulate the activity of the Akt/PKB pathway, JNK, mTOR, ERK, and β-catenin^11–13^. Aldehyde dehydrogenase and ATP-binding cassette superfamily transporters such as ABCG2 are also elevated in CD133^+^ liver CSCs^14^. Additionally, CD133^+^ LCSCs can promote angiogenesis via the regulation of the production of IL-8, VEGF, and MMP-2^15^. Current studies have indicated that CD133 is expected as a novel target to overcome chemo-resistance in HCC^10^.

We has developed an automated imaging platform, which systematically analyzes cytotoxic effects in cell culture based on a state-of-the-art fluorescence imaging platform and high-end image analysis technology to accurately ascertain the cytotoxic events in HCC cells. Further, it is also in our full capacity to monitor and analyze the cellular phenotype of individual cell types or functional cellular states implementing dedicated quantitative image analysis algorithms^16^.

In this study, we aimed to develop LCSC-specific drugs that could induce cell death in LCSC, while minimizing the damage to normal hepatocytes, in a mixed cell culture system containing hepatocytes, LCSC and HCC cells. To this end, we developed image-based approaches to quantify complex HCC cell populations, in terms of cellular phenotype and global cell population evaluations that could be used for drug discovery for liver cancer therapy. Subsequently, we performed screening to identify compounds that specifically alter the properties of the LCSC in HCC-mixed population.

## Materials and Methods

### Cell lines and culture conditions

For human immortalized hepatocyte cell line, Fa2N-4 cells were used in this study. FaN-4 cells and culture medium were obtained from Xenotech (Lenexa, KS, USA), cells were plated in collagen-coated plates first for 3–6 hr with serum-containing plating medium (K4000), and was changing with supporting culture medium (K4100.X). For human hepatocellular carcinoma cell lines, huh7.5-RFP-NLS-IPS reporter cell line (Huh7.5-RFP), Huh7 and Hep3B were examined in this study. Huh7.5-RFP was kindly provided by Dr. Marc Windisch (Institut Pasteur Korea). Huh7 and Hep3B were purchased from the Korean Cell Line Bank. All HCC cell lines were cultured in Dulbecco’s minimal essential medium (DMEM; Welgene, Korea, LM001-05) supplemented with heat-inactivated 10% fetal bovine serum (FBS; Gibco, Gaitherburg, MD, USA) and 100U/ml Penicillin and 100 µg/ml Streptomycin (P/S; Gibco) at humidified 37 °C incubator under 5% CO_2_.

### HTS/HCS screening

Mixed culture model of Huh7.5-RFP and Fa2N-4 was used in CD133-target screening. Fa2N-4 and Huh7.5-RFP cells were seeded together at a density of 1.5 × 10^3^ cells/well and 0.8 × 10^3^ cells/well respectively in 384-well plate (Greiner Bio-One, Monroe, NC, USA; 781091). After 16 hr incubation, compounds were treated at final concentration of 10 µM in 0.5% dimethyl sulfoxide (DMSO) (v/v) (Sigma-Aldrich, St Louis, MO, USA). 10 µM of sorafenib (Santa Cruz Biotechnology, Dallas, TX, USA) and 0.5% DMSO were used as positive and negative control, respectively. A library collection of 3,280 compounds were assembled from LOPAC (St Louis, MO, USA), Prestwick Chemical (Washington, DC, USA), and Enzo Life Sciences (FDA-approved compound library; Farmingdale, NY, USA). For the testing of compounds, 1 µl of each compound was transferred into an intermediate 384-well polypropylene plate (Greiner Bio-One) using a liquid handler (Apricot Personal Pipettor; Apricot Design, Covina, CA, USA). The compounds were mixed with 49 µl/well of complete medium. Subsequently, 10 µl of compound was dispensed into each well of a 384-well assay plate for 48 hr. The plates were then incubated at 37 °C in a humidified atmosphere of 5% CO_2_. After 48 hr incubation, cells were fixed with 4% paraformaldehyde (PFA; Sigma-Aldrich) and reacted with mouse monoclonal anti-human CD133/1 (AC133, 130-090-422, 1:100; Miltenyi Biotec, Bergisch Gladbach, Germany) for 16 hr at 4 °C and washed with Dulbecco’s phosphate-buffered saline (DPBS; Welgene) for 10 min, and were incubated for 2 hr at room temperature (R.T.) with goat anti-mouse Alexa Fluor 488 secondary antibody (Invitrogen, Eugene, OR, USA). After three times washing with DPBS, cells were stained with Hoechst 33342 (Invitrogen) for detecting nucleus. Cell images were obtained by Operetta High Content Screening (HCS) System (Perkin Elmer, Waltham, MA, USA) and analyzed by Harmony 3.5.1 high content imaging and analysis software (Perkin Elmer). The compounds that >80% of cell survival and <5% of CD133 positive cells (Cancer stem cells) were selected. Z’ factor was calculated by excel with the following formula: 1 − [{3 × (Standard Deviation of Batch of Compounds Tested) + 3 × (Standard Deviation of the 100% Inhibition Control)}/(Mean of the Compound Batch Tested − Mean of the 100% inhibition Control)].

### Cell sorting

Huh7.5 cells were sorted by fluorescence-activating cell sorting (FACS; BD Biosciences, Franklin Lakes, NJ, USA) using mouse anti-human CD133/1 (AC133) conjugated with VioBright FITC (130-105-226. 1:11; Miltenyi Biotec). The cells were treated with 0.05% trypsin (Gibco) and harvested, washed twice with DPBS added with 5% FBS and re-suspended in DPBS added with 10% FBS with mouse anti-human CD133/1 (AC133) conjugated with VioBright FITC for 30 min at 4 °C in dark. The cells were washed twice with pre-cooled DPBS and centrifuged at 1,200 rpm at 4 °C, and cells were sorted by FACS. CD133^−^ and CD133^+^ HCC were cultured in DMEM supplemented with 10% heat-inactivated FBS and 1% P/S.

### Dose-response curve (DRC)

Huh7.5 cells or Huh7.5-CD133^+^ cells and Huh7.5-CD133^−^ cells were seeded at a density of 2,000cells/well in 384-well plate, and hit compounds were treated and incubated for 48 hr. The concentration of hit compounds are from 10 µM to 39.06 nM (2-fold dilution from 10 µM) in 0.5% DMSO (v/v). After 48 hr, cells were fixed with 4% PFA for 10 min at R.T., and washed twice with DPBS. For nucleus staining, Hoechst 33342 were used for staining. To analyze the enough cells (>1,000), five image fields were captured and collected from each well, starting at the center. All of the image analysis was performed using HCS system and Harmony software. Cell count were calculated and normalized to control (0.5% DMSO).

### Polyacrylamide gel electrophoresis (PAGE) and western blot analysis

Proteins in cells were harvested in lysis buffer (3 M, Maplewood, MN, USA) through incubating for 30 min in ice and centrifuged at 13,200 rpm for 10 min at 4 °C. The supernatant were transferred to new 1.5 ml tube (Corning, Corning, NY, USA), and the samples were mixed with 5x sample buffer (BioSolution, Seoul, Korea). After boiling for 10 min, equal same amounts of protein (10–30 µg/well) were separated on 8 or 10% SDS-PAGE gels depending on the protein size. After electrophoresis, the proteins were transferred onto a nitrocellulose (NC) membrane (Pall, Port Washington, NY, USA), and blocked with 5% skim milk (BD Bioscience) for 30 min at R.T. After blocking, the NC membranes were incubated with mouse anti-human CD133/1 (AC133, 130-090-422, 1:100; Miltenyi Biotec, Bergisch Gladbach, Germany), rabbit anti-human alpha 1 fetoprotein (AFP, ab169552, 1:1000; Abcam, Cambridge, UK), rabbit anti-human EpCAM (ab71916, 1:1000; Abcam), rabbit anti-human CD44 (ab51037, 1:1000; Abcam) and mouse anti-human β-actin (A5441, 1:10,000; Sigma-Aldrich) for 16 hr at 4 °C. After washing, the blots were incubated with anti-mouse IgG, horseradish peroxidase-conjugated secondary antibody (7076, 1:10,000; Cell Signaling Technology, Danvers, MA, USA) or anti-rabbit IgG, horseradish peroxidase-conjugated secondary antibody (7074, 1:10,000; Cell Signaling Technology) for 1 hr. After washing, the specific bands were visualized by enhanced chemiluminescence (ECL; Thermo Fisher Scientific, Waltham, MA, USA) and recorded on X-Omat AR films (Estman Kodak Co., Rochester, NY, USA). β-actin was used as control of each samples.

### Formation of live cancer stem cell (LCSC) spheroids

Huh7.5-RFP cells were seeded in 96-well round bottom ultra-low attachment microplates (Corning) at a density of 1 cell/well in LCSC media. LCSC media is composed of DMEM/F12 (10565-018, Gibco) added with 1x B27 (Invitrogen), 20 ng/ml basic fibroblast growth factor (bFGF; Invitrogen), 20 ng/ml epidermal growth factor (EGF, Invitrogen), 25 µg/ml insulin (Sigma-Aldrich) (LCSC media) and cultivated at humidified 37 °C incubator under 5% CO_2_ for 5 days. After 5 days, homogenous and singular spheroid was transferred to new 96-well round bottom ULA microplates and treated with 4 hit drugs, sorafenib (positive control), and 0.5% DMSO (negative control) for 7 days. Bright-filed images were obtained everyday with HCS System.

### Flow cytometry analysis

For counting the CD133^+^ cells after treating with 0, 10, 50, 100 µM of oxytetracycline in Huh7 and Hep3B for 48 hr, cells were treated with 0.05% trypsin and washed twice with DPBS supplemented with 5% FBS and re-suspended in DPBS supplemented with 10% FBS with mouse anti-human CD133/1 (AC133) conjugated with VioBright FITC for 30 min at 4 °C in dark. After washing with DPBS twice, cells were analyzed by flow cytometry.

### Protein synthesis study

To investigate the protein synthesis inhibition of oxytetracycline in HCC cells, Click-iT AHA Alexa Fluor 488 Protein Synthesis HCS Assay kit was used (C10428, Invitrogen). The assay was performed following the instructions of kits. Briefly, 2 × 10^3^ Huh7 and 2.5 × 10^3^ Hep3B cells/well were seeded in 384-well plates and incubated at humidified 37 °C incubator under 5% CO_2_ overnight. Cells were treated with 0, 10, 50, or 100 μM oxytetracycline for 48 hr. After removing the drug-containing medium, the cells were incubated for 30 min in a methionine-free medium (21013, Gibco) with 50 μM of L-homopropargylglycine-an amino acid analog of methionine containing an alkyne moiety, which is incorporated into proteins during active protein synthesis. Afterwards, the cells were fixed with 4% PFA for 10 min at R.T. in dark and washed with DPBS twice, and permeabilized with 0.5% Triton X-100 (Sigma-Aldrich) in DPBS and incubated for 20 min at R.T. The cells were then incubated with Click-it reaction cocktail (containing Alexa Flour 488 conjugated alkyne) for 30 min at R.T. in dark. The cocktail was removed and washed with Click-iT reaction rinse buffer. The images obtained using HCS system with excitation/emission wavelengths of 386/535 nm.

### Protein stability assay

To examine the CD133 stability by oxytetracycline, Huh7 and Hep3B cells were treated with 0, 10, 50 or 100 µM of oxytetracycline, and after 24 hr, 0 or 30 µg/ml cycloheximide (CHX) was treated for further 24 hr. After 48 hr from oxytetracycline treatment, cells were trypsinized and prepared for western blot for CD133 antibody.

### Tumor Xenograft in nude mice

Huh7.5 (1 × 10^6^ cells) with 95% viability was injected subcutaneously into the hind leg of 6-well-old BALB/c athymic nude mice (Central Lab. Animal Inc., Seoul, Korea). When tumors reached a volume of 150–200 mm^3^, mice were randomly divided to 6 groups as follows: (1) the tumor control group (0.9% saline), (2) the oxytetracycline group (200 mg/kg, 5 days), (3) Sorafenib group (10 mg/kg, 5 days). Each group is composed of three mice. The volume of tumors was calculated using the following formula: (LxI^2^)/2, where L = tumor length and I = tumor width. Dimensions were determined using calipers. Oxytetracycline and sorafenib were dissolved in saline (0.9% NaCl) and injected intraperitoneally (i.p.) and oral, respectively, daily for 5 days. The mice were sacrificed for immunohistochemistry after 10 days from drug injection. The animal protocol was approved by the institutional animal care and use committee of ASAN medical center and conducted strictly in accordance to the national institute of health guide for the care and use of laboratory animals.

### Histological analysis

Tumor tissues were fixed in 4% PFA, cut into 4 µm paraffin-embedded sections. For immunohistochemistry, after deparaffinization and dehydration, antigen retrieval was performed by boiling the sections in 10 mM citric acid buffer (pH 6.0) for 15 min. Goat anti-human CD133/1 (AC133) was used for detecting CD133 protein in tissues, and detected with DAB peroxidase (HRP) substrate kit. The animal protocol was approved by the institutional animal care and use committee of ASAN medical center and conducted strictly in accordance to the national institute of health guide for the care and use of laboratory animals.

### Reverse transcription-polymerase chain reaction (RT-PCR) and real-time polymerase chain reaction (Real-time PCR)

Total RNA was isolated from cells using TRIzol(Invitrogen) according to the manufacturer’s instructions. The reaction mixture were comprised of RT buffer (Bio Basic, Amherst, NY, USA), dNTP solution (Bio Basic), RNasin inhibitor (Promega, Madison, WI, USA), oligo (dT)^15^ primer (Bioneer), total RNA, and M-MLV reverse transcriptase (Invitrogen). The reaction mixtures were incubated at 37 °C for 1 hr and the transcription reaction was terminated by heating the mixture to 95 °C for 5 min and then rapidly cooling it on ice. The number of PCR cycles used was 30 for all reactions. The PCR products were then separated by 2% agarose gel electrophoresis and visualized with 5x loading star (Dynebio, Seoul, Korea).

For real-time PCR, the mixture composed of cDNA, SYBR Green master mix (Applied Biosystems, Waltham, MA, USA), primers and DEPC, were performed using a StepOnePlus real-time PCR system (Applied Biosystems). The reactions were incubated in a 96-well optical plate at 95 °C for 10 min, followed by 40 cycles of 95 °C for 15 sec and 60 °C for 1 min. The threshold cycle (CT) is defined as the fractional cycle number at which the fluorescence passes the fixed threshold. CT values were normalized to GAPDH, and calculated according to the mathematical model R = 2−ΔΔCT, where ΔCT = CT_target gene_ − CT_GAPDH_, and ΔΔCT = ΔCT_test_ − ΔCT_control_. All real-time PCR was performed in triplicates, and the data are presented as the mean ± SD. All primers were designed and purchased from Bioneer.

### Statistical analysis

All experiments were performed at least three times. The results are shown the mean ± standard deviation (SD). Statistical analysis was performed using Student’s t-test.

## Results

### Non-target based high throughput screening for compounds that regulate CD133^+^ hepatocellular carcinoma

**Figure 1:**
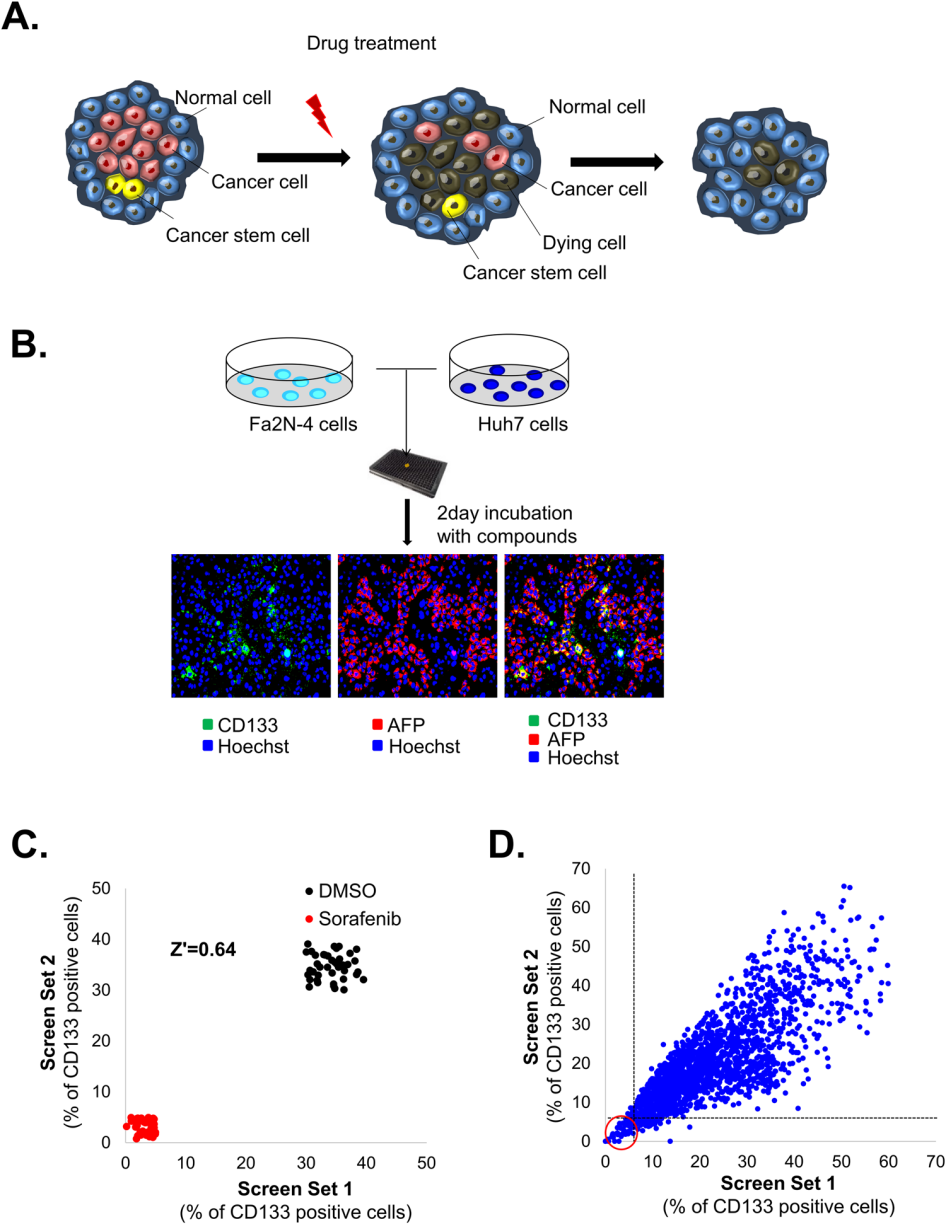
Hepatocellular carcinoma (HCC)-mixed culture system for liver cancer stem cells (LCSCs)-targeting drug screening. (**A**) Schematic illustration of HCC-mixed culture system for LCSC-targeting drugs. The HCC-mixed culture system, which is composed of HCC cells, CSCs and normal hepatocyte, can be used to screen for LCSC-specific targeting drugs. (**B**) Image-based phenotypic screening for identifying the CSC targeting drug discovery. Fa2N-4 cells and Huh7.5 cells were seeded together in each well of 384-well plates overnight, and 10 μM of compounds were treated for 48 hr. After 48 hr, cells were stained with CD133 to analyze the each population (Cancer cells, CSCs, and normal hepatocyte). (**C**,**D**) Pilot screen results. (**C**) The scatterplot analysis shows positive (red: 10 μM sorafenib) and negative (black: 0.5% DMSO) control among the validation plates. Dots represent each sing well tested. (**D**) A total 3,280 compounds were screened at a concentration of 10 μM validation plates. Dots represent single tested wells. Dots in red circle indicate the primary hit compounds. The correlation coefficient (r^2^ = 0.6398) was calculated by using the obtained optimal number of components. All images were obtained using the Operetta High Content Screening (HCS) system.

In previous study, we characterized CSCs in primary HCC and identified CD133 as a CSC cell-surface marker^7^. To identify drugs inducing cell death in LCSC while minimizing the damage on hepatocytes, we set up well-defined HCC-mixed culture system in 384-well plate for image-based phenotypic screening using Fa2N-4 cells (immortalized hepatocyte line) and Huh7.5 cells [Fig. 1A]. In this study, we used Huh7.5-RFP-NLS-IPS reporter cell line, which expressed RFP and AFP simultaneously with the same localization, for improvement of the efficiency of the drug screening process after drug treatment [Supplementary Fig. 1]. Subsequently, we performed screening to identify compounds that specifically alter the properties of the AFP^+^/CD133^+^ cells as LCSC. Compounds that have such effects would be validated by assessing their effects using immunostaining of the selected markers (AFP^+^/CD133^+^) for distinguishing the CSC populations. Compounds were screened at an initial concentration of 10 uM with a readout looking at a decreased LCSC population but not HCC (AFP^+^/CD133^−^), hepatocyte (AFP^−^/CD133^−^) population [Fig. 1B]. Positive and negative controls would be 10 μM sorafenib and 0.01% DMSO, respectively. A Z’ factor of 0.64 indicated that the assay was reliable [Fig. 1C].

We screened 3,280 compounds selected from compounds libraries (including LOPAC, Prestwick and Enzo (FDA-approved compound)) for drug repositioning in duplicate to confirm the reproducibility of observed effects. Then follow up dose response studies performed to quantify the potency of selected hits. A pearson correlation coefficient of 0.6398 for replicate screens indicated that the assay was reliable [Fig. 1D]. 13 compounds as the primary hits that significantly inhibited AFP^+^/CD133^+^ population in HCC-mixed culture system [Table 1].

**Table 1:** List of primary hit compounds.

	Name of compounds	Mechanism of action
1	5′-Amino-5′-deoxyadenosine p-toluenesulfonate salt	Adenosine analog.
2	trans-Azetidine-2,4-dicarboxylic acid	mGluR1 and mGluR5 metabotropic glutamate receptor agonist
3	Chlormethiazole hydrochloride	GABA(A) agonist; glycine receptor modulator
4	beta-Chloro-L-alanine hydrochloride	Alanine aminotransferase inhibitor
5	1,10-Diaminodecane	Inverse agonist at the polyamine recognition site of the NMDA glutamate receptors
6	LY-294,002 hydrochloride	Specific phosphatidylinositol 3-kinase (PI3K) inhibitor.
7	Pentamidine isethionate	NMDA glutamate receptor antagonist
8	PAPP	Selective 5-HT1A serotonin receptor agonist
9	Mebendazole	Glucose transport inhibitor
10	Oxytetracycline	Ribosomal protein synthesis inhibitor
11	Fusidic acid sodium salt	Protein synthesis inhibitor GTPase coupled
12	(S)-(-)-Atenolol	Adrenergic receptor antagonist
13	Tetramisole hydrochloride	Alkaline phosphatase inhibitor

### Confirmation of Hit compounds

**Figure 2:**
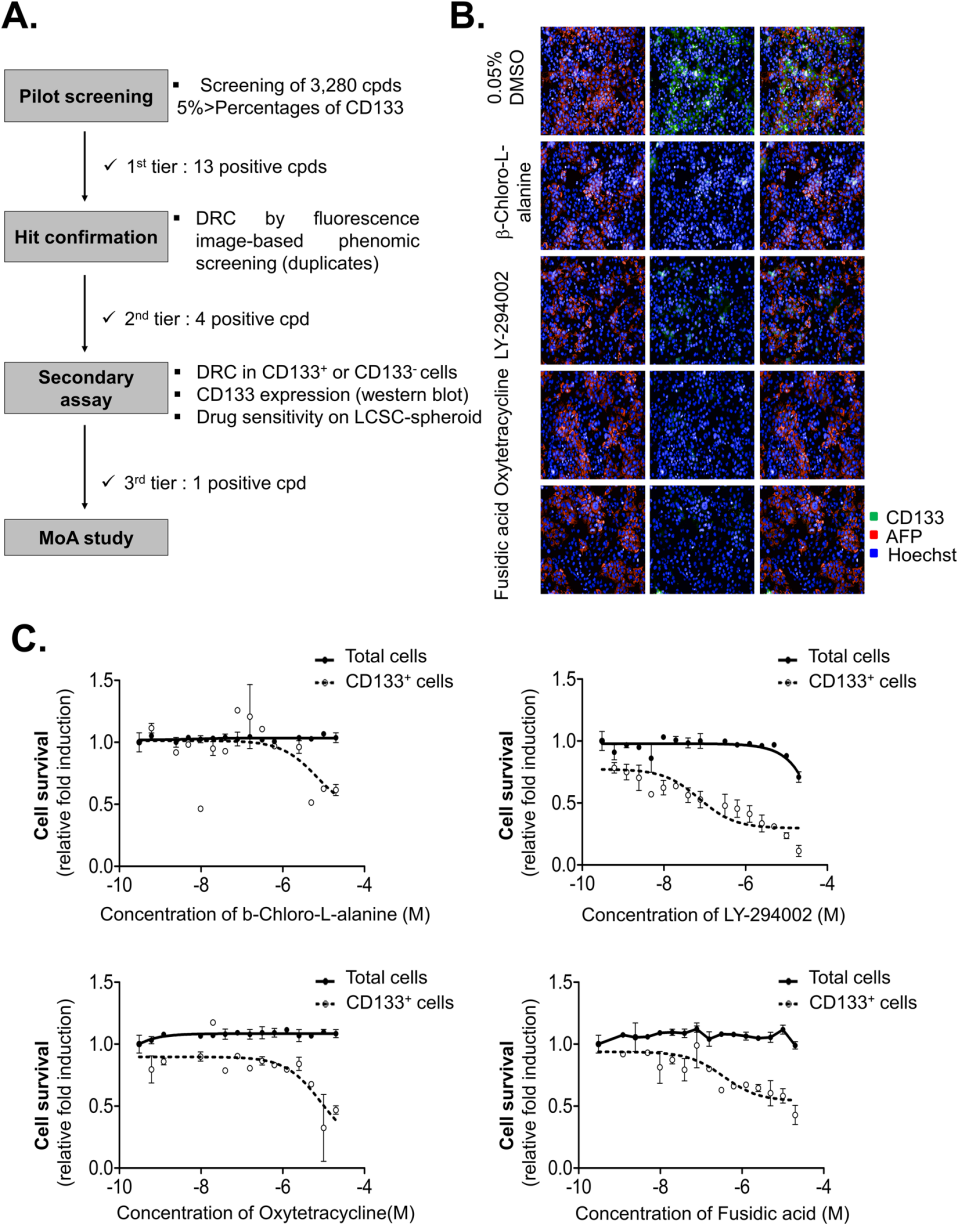
Four hit compounds from HCC-mixed LCSC-targeting screening. (**A**) Procedure of HCC-mixed LCSC-targeting screening. 13 Compounds, which decreased CSC population <5% of CD133, were selected from pilot screening. HCC-mixed cells were treated with 13 compounds from pilot screening at indicated concentration, and analyzed cell survival of each population through image-based phenomic screening (p < 0.05). From dose response curve (DRC), 4 positive compounds were selected. These compounds were tested with secondary assay, which are DRC in CD133-sorting cells (CD133^+^, CD133^−^ cells), CD133 expression level, and drug sensitivity on LCSC-spheroid. From these assay, 1 positive compound was finally selected, and mode of action (MOA) of final compound was studied. (**B**) Images of cells treated with 4 hit compounds (ß-Chloro-L-alanine, LY-294002, Oxtetracycline and Fusidic acid). (**C**) Cell survival of total cells and CD133^+^ HCC at indicated concentration of 4 hit compounds. All images were obtained HCS system and analyzed by Harmony software. Data are shown as the mean and standard deviation (SD) of triplicate experiments.

Through the pilot screening, we found LCSC population-specific drugs that significantly induced cell death in HCC (AFP^+^/CD133^+^) cells while minimizing the damage to hepatocytes (AFP^−^/CD133^−^). To verify hits from the primary screen, we followed procedure described in Fig. 2A.

13 representative candidates were retested at the original screen concentration of 10 μM; of these, 4 compounds (28.6%), which are β-Chloro-L-alanine hydrochloride (alanine aminotransferase inhibitor), LY-294,002(PI3K inhibitor), oxytetracycline (ribosomal protein synthesis inhibitor) and fusidic acid (protein synthesis inhibitor GTPase coupled), showed significant inhibition activity of AFP^+^/CD133^+^ HCC population without damage on hepatocytes [Fig. 2B]. There is no similar structure of compounds among hits compounds.

To determine specificity to CD133^+^ HCC cells, we detected dose-response of 4 hit compounds in Huh7.5 cell lines. Indeed, these hit compounds selectively suppress the CD133^+^ population without decreasing of total cell number [Fig. 2C].

To confirm inhibitory effects of 4 hit compounds on CSC properties, we proceeded certain secondary assays, which are dose-response in CD133^+^ or CD133^−^ cells, evaluation of CD133 expression, drug sensitivity on LCSC-spheroid [Fig. 2A].

**Figure 3:**
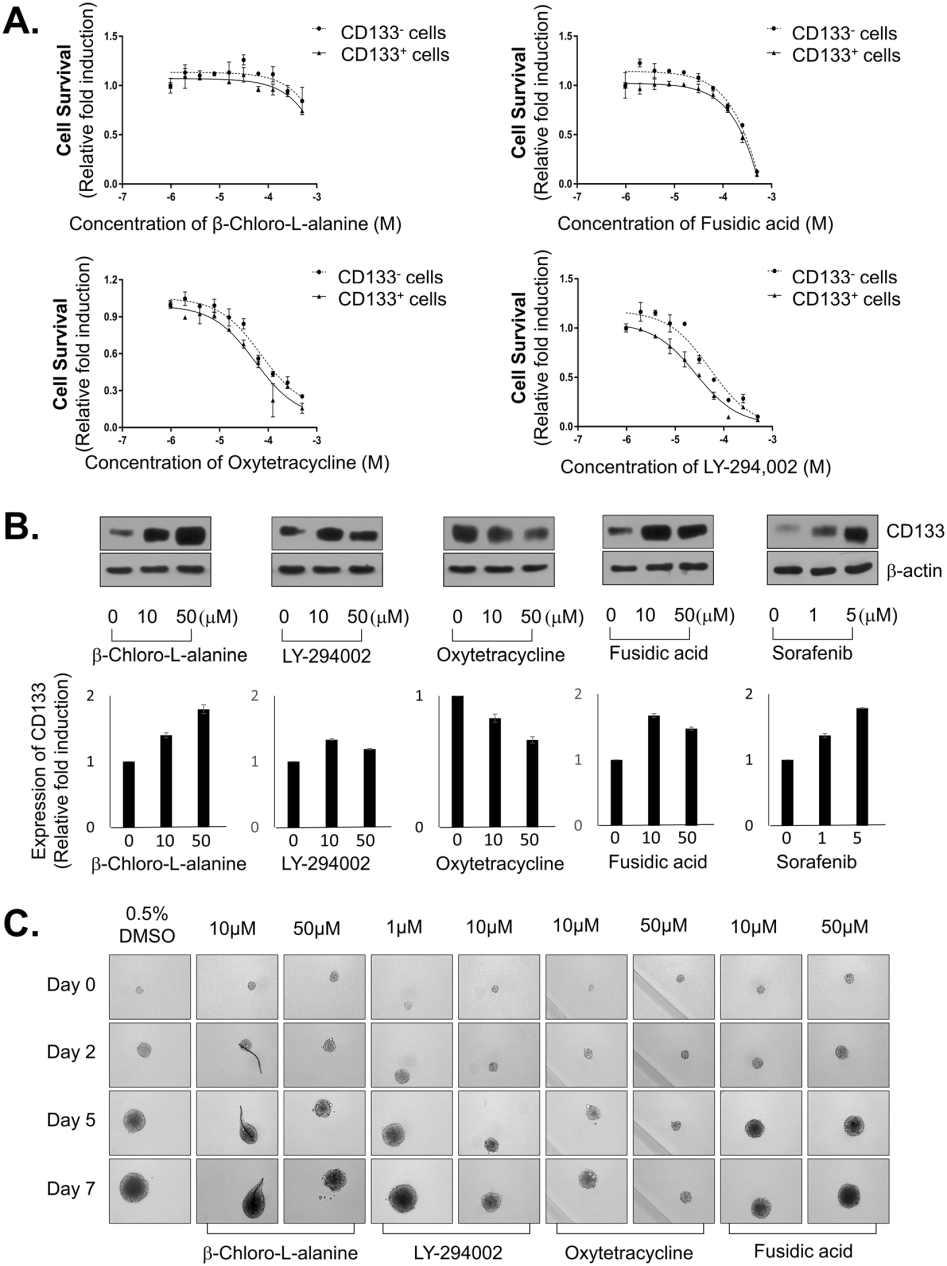
Suppressing LCSC properties of 4 hit compounds. (**A**) Cell survival of CD133^−^ and CD133^+^ HCC cells treated with 4 hit compounds. CD133^−^ and CD133^+^ cells were sorted from Huh7.5 cells, and treated with 4 hit compounds at indicated concentration for 48 hr. Cells were stained with Hoechst33342 for nucleus, and nucleus were counted and analyzed. (**B**) CD133 expression level after treating with 4 hit compounds in Huh7.5 cells. Huh7.5 cells were treated with indicated concentration of 4 hit compounds for 48 hr, and the CD133 expression levels were analyzed through western blot (upper panel), and the intensity of bands were analyzed (lower panel). (**C**) Spheroid formation capacity of LCSCs treated with 4 hit compounds. Huh7.5 cells were seeded at a density of 1 cell/well in LCSC-permission media and cultivated for 5 days, and treated with 4 hit compounds of indicated concentration for 7 days. The images were obtained using HCS system. Data are shown as the mean and standard deviation (SD) of triplicate experiments. Scale bar = 200 μm.

First, we isolated CD133^+^ and CD133^−^ HCC cells from Huh7.5 cells and number of cells was detected after treatment with hit compounds in CD133^+^ HCC and CD133^−^ HCC cells. Among 4 hit compounds, LY-294,002 and oxytetracycline remarkably exhibited more sensitive effect in CD133^+^ HCC than CD133^−^ HCC significantly at high concentrations [Fig. 3A]. Next, we estimated CD133 expression by western blot analysis after treatment of 4 hit compounds and sorafenib, which is uniquely approved for the treatment of patients with advanced HCC. However, unlike our predictions, most of the compounds include sorafenib could not inhibit the expression of CD133. Rather, most of the compounds induced increasing of the expression of CD133. Intriguingly, oxytetracycline could decrease of the expression of CD133 [Fig. 3B].

We further investigated whether 4 hit compounds affected capacity of LCSC spheroids formation, because CSCs are enriched in non-adherent spheroids of breast, colon, and liver cancer cells. In line with our western blot analysis results, we found that oxytetracycline the most strongly attenuated the strong capacity of LCSC spheroids formation among hit compounds [Fig. 3C].

Altogether, those data indicated that oxytetracycline selectively suppressed the LCSC (AFP^+^/CD133^+^ HCC) population and LCSC properties and suggested oxytetracycline as an appropriate drug to prevent HCC recurrence and to overcome chemoresistance.

### Oxytetracycline suppresses CD133 expression by decreasing of protein stability of CD133

**Figure 4:**
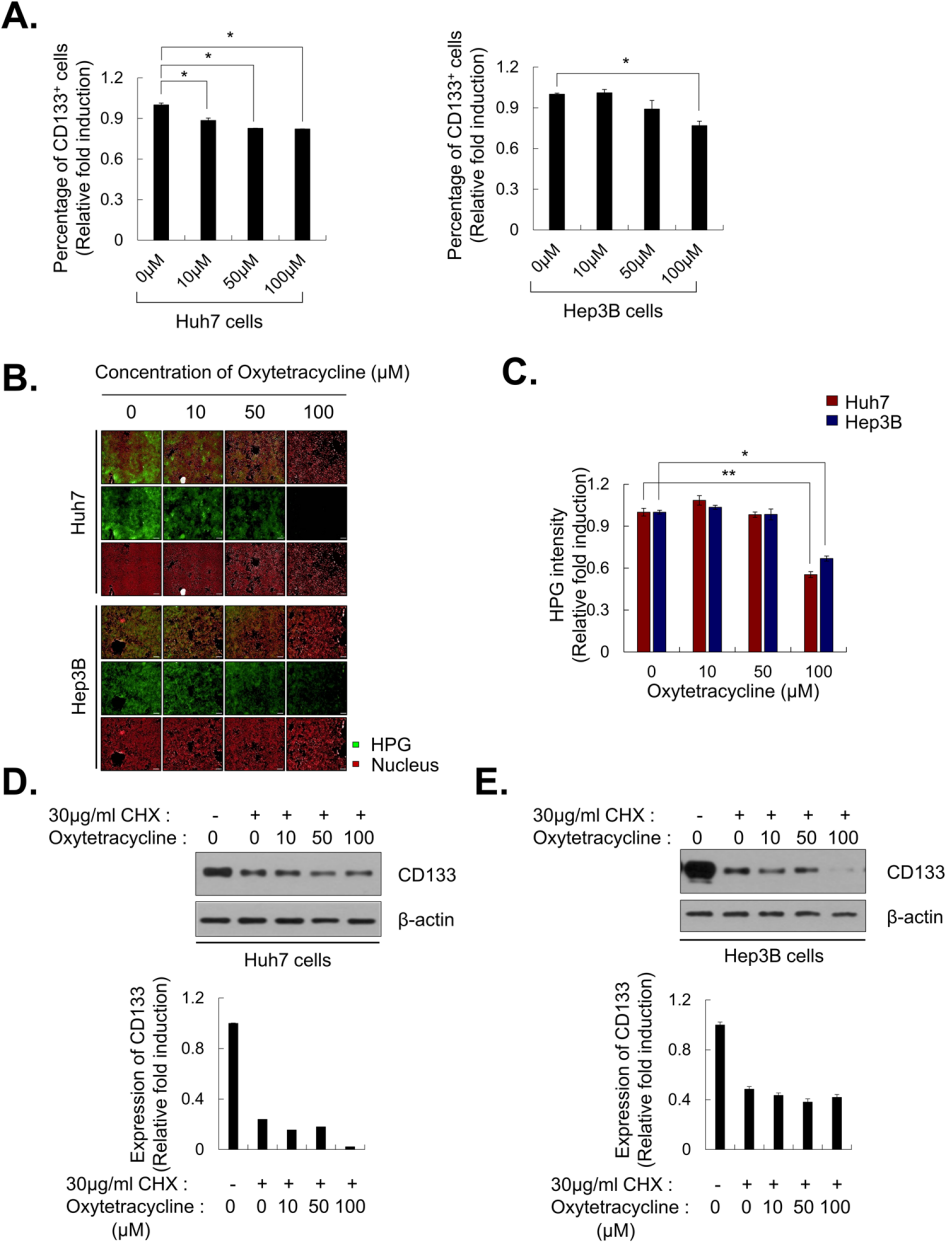
Decrease of CD133 levels by oxytetracycline. (**A**) CD133 expression level by oxytetracycline treatment in Huh7 and Hep3B cells. Cells were treated with indicated concentration of oxytetracycline for 48 hr, and CD133 expression levels were examined with FACS analysis. (**B**,**C**) Inhibition of protein synthesis by oxytetracycline. (**B**) HPG-labeled proteins were monitored by treatment of oxytetracycline in Huh7 and Hep3B cells. Nuclei were detected by staining with Hoechst 33342, and (**C**) HPG intensity was analyzed. (**D**,**E**) Degradation of CD133 protein by oxytetracycline. Huh7 cells (**D**) and Hep3B cells (**E**) were treated with oxytetracycline with indicated concentration and one day after 30 μg/ml cycloheximide was treated for 24 hr. After 48 hr from oxytetracycline treatment, cells were prepared for western blot. All images were obtained and analyzed with HCS system. Data are shown as the mean and standard deviation (SD) of triplicate experiments. Scale bar = 200 μm. *p < 0.05, **p < *0.005*.

Once we identified oxytetracycline, we sought to search potential mode of actions in LCSC. To ascertain effects of oxytetracycline on CD133^+^ HCC, we estimated CD133^+^ HCC population in Huh7 cells and Hep3B cells, which have plenty of CD133^+^ HCC population among HCC cell lines relatively, after treatment of oxytetracycline by FACS analysis. Oxytetracycline sufficiently suppressed the CD133^+^ HCC population in Huh7 cells and Hep3B cells [Fig. 4A].

The ability to detect and characterize newly synthesized proteins, changes in protein expression, or protein degradation resulting from drug treatments is an important parameter in cytotoxicity measurements. Specially, because oxytetracycline is famous antibiotics and ribosomal protein synthesis inhibitor, we monitored the inhibition of the protein synthesis with oxytetracycline using Click-iT HPG (L-homopropargylglycine). The dose-dependent decreasing of HPG-labeled proteins was monitored by treatment of oxytetracycline in Huh7 cells and Hep3B cells [Fig. 4B,C].

Next, we examined whether the protein stability of CD133 were affected by treatment of oxytetracycline, because it could suppress the LCSC properties, CD133^+^ HCC population and CD133 expression. The half-life of CD133 protein following treatment with cycloheximide was decreased by oxytetracycline when compared with 0.05% DMSO in Huh7 cells and Hep3B cells in a dose-dependent manner [Fig. 4D,E] and time-dependent manner [Supplementary Fig. 2].

We investigated whether treatment of oxytetracycline was associated with alteration in the display of different kinds of CSC markers and found no changing in the expression of CSC markers (EpCAM, AFP, CD44) after treatment of oxytetracycline [Supplementary Fig. 3]. The mRNA level of CD133 as well as stem cell-relative makers (such as SOX2, c-Myc, SCF, Oct4, Nanog) in Huh7 cells and Hep3B cells that were treated oxytetracycline did not change [Supplementary Fig. 4].

After treating tetracycline and doxycycline, functionally and structurally similar with oxytetracycline, we also observed expression of CD133 in Huh7 cells and Hep3B cells. Treatment of tetracycline and doxycycline didn’t display alteration of CD133 expression [Supplementary Fig. 5]. Taken together, these results suggests that oxytetracycline can alter LCSC properties through decreasing of CD133 protein stability in HCC cells.

### Oxytetracycline have the therapeutic efficiency in CD133+ HCC population

**Figure 5:**
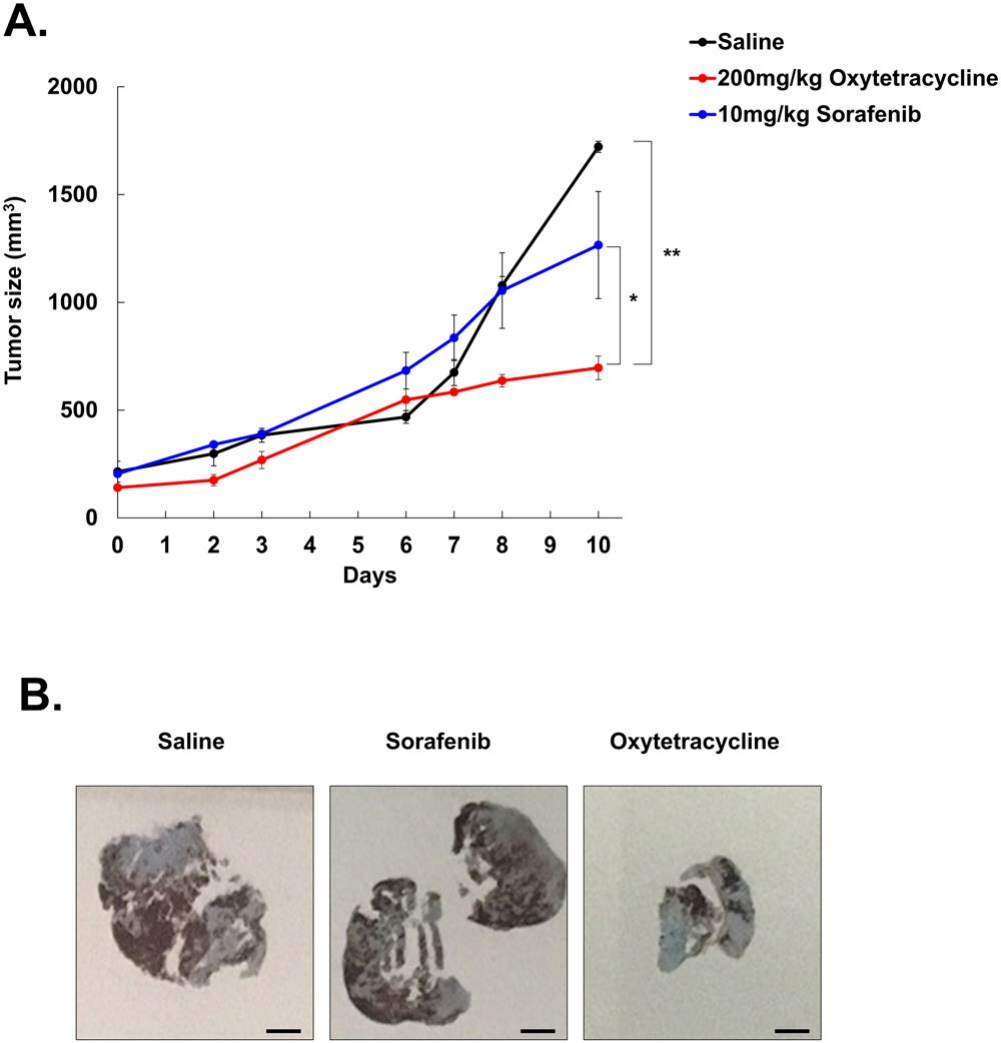
Decrease of tumor size and CD133^+^ population mouse model. (**A**) Tumor size of indicated groups. When the tumor volume was reached 150–200 mm^3^, mice were randomized to 3 groups (Saline, Sorafenib, Oxytetracycline) were injected for 5 days. The tumor volume in the indicated mouse groups was examined every 2 days for evaluating the compounds effects. (**B**) Immunohistochemistry of CD133 in Huh7.5 tumor tissues of indicated groups in xenograft mice. Data are shown as the mean and standard deviation (SD) of five animals in each group. *p < 0.05, **p < *0.005*.

In previous study, we found that the CD133^+^ cells displayed higher tumorigenicity and faster tumor growth compared with the CD133^−^ cells and suggesting that CD133^+^ cells have the ability to initiate HCC tumor formation^17^. Hence, we transplanted 5 × 10^4^ cells of the CD133^+^ Huh7 populations into NOD/SCID mice to analyze capability of the LCSC suppression by oxytetracycline. Administration of oxytetracycline inhibited tumor growth without loss of body weight [Supplementary Fig. 6], versus mice treated with sorafenib, which displayed a subtle decreasing of tumor growth [Fig. 5A].

Next, we explored whether oxytetracycline can reduce CD133^+^ cells specifically in HCC. Immunohistochemical analysis showed that oxytetracycline sufficiently diminished the CD133^+^ cell population in Huh7 tumor cells relative to treatment of sorafenib *in vivo* [Fig. 5B].

These results showed that oxytetracycline can decrease CD133^+^ HCC cells by reducing stabilization of CD133 expression. Taken together, our data suggest that treatment with oxytetracycline could suppress CD133^+^ HCC population and LCSC properties via destabilization of CD133 in CD133^+^ HCC cells, thereby promoting the therapeutic efficacy of oxytetracycline in human liver carcinomas.

## Discussion

In previous our study, we developed a image-based phenotypic screening system *in vitro* to identify hepatocellular carcinoma (HCC)-specific drugs in mixed culture of HCC cells with hepatocytes^16^. The dream goal is discovery of drug candidates targeting selectively the cancer stem cells (CSCs) of HCC. CD133^+^ HCC may be a vulnerable “Achilles heel” of HCC^18^. Therefore, here, we have developed the mixed HCC cell population using HCC cells and hepatocytes that are state of the art, comparable with the mixed culture system published in the literature.

CD133^+^ cells exhibit the capacity for self-renewal, proliferation, differentiation, and tumor-formation^19,20^. We characterized CSCs in primary HCC and identified CD133 as a CSC cell-surface marker and elucidated CD133 facilitates CSC-like properties by stabilizing EGFR-AKT signaling in HCC^21^. Additionally, we elucidated that oxidative stress elevate expression of CD133 in HCC and increased CD133 expression facilitate the capacity for ROS defense. Based on these studies, we suggested that overexpression of CD133 have a critical roles in chemoresistance during liver cancer therapy^19,22^. On the other hand, elimination of CD133 expression diminished the capacity for defense against ROS and chemoresistance in HCC. Moreover, CD133 depletion destabilized EGFR by augmenting EGFR internalization and thus inhibited CSC-like properties. As such, CD133 can put HCC in a more severe environment in many directions and control of CD133 expression is very important to maintain the CSC-like properties^23^. The CD133^+^CSCs may be resistant to hypoxic conditions and proliferate for survival. In a related study, CD133^+^ glioma cells were increased through HIF-1α activation under hypoxic condition, which may also be mediated by AKT of the ERK1/2 pathway^24^. Additionally, CD133 likely exhibit function of CSC through not only the activation of signal molecules such as PI3K-Akt, Src-Focal adhesion kinase and EGFR by direct interaction^21,22,25^, but also control of cancer metabolism^26^. Despite active research on the characteristics of CD133^+^ LCSCs in HCC, the molecular mechanisms of CD133 have not been fully elucidated and inhibitors of CD133 have not been developed yet.

Hence, we aimed to use non-target based high throughput screening (HTS) approach to identify compounds inducing cell death in CD133^+^ HCC while minimizing the damage on hepatocytes [Fig. 1A].

In primary screening using FDA-approved compounds for drug repositioning, 13 compounds were identified as hits compounds that appear to fulfill the criteria of selectivity [Table 1]. Through hit confirmation and secondary assay, we finally identified one positive compound, which is oxytetracycline that induced cell death in CD133^+^ HCC while minimizing the damage on hepatocytes [Figs 2, 3].

Oxytetracycline, discovered in the early 1950s by Alexander Finlay at Pfizer (Groton, CT, USA), was the first member of the tetracycline group. Oxytetracycline is produced by *Streptomyces rimosus* and it was isolated in Terre Haute, IN, USA, and therefore called terramycin^27^. The oxytetracycline commonly plays a role as antibiotic that acts by inhibiting protein synthesis in bacteria. Moreover, oxytetracycline suppresses mucus production and inflammation in human respiratory epithelial cells and attenuates allergic airway inflammation in mice via inhibition of the NF-κB pathway^27,28^. Recently, some reports showed that oxytetracycline also have anticancer effects as mitochondrial metabolism-interfering agent in certain types of cancer cell lines such as A549, Hela and DU145 cells^29,30^.

FDA-approved antibiotics were suggested to eradicate CSCs, in 12 different cancer cell lines, across 8 different tumor types (breast, DCIS, ovarian, prostate, lung, pancreatic, melanoma, and glioblastoma (brain))^28,31^.

In this study, we found novel functions of oxytetracycline in terms of anticancer efficacy. The oxytetracycline suppresses stemness [Fig. 3C] through inducing of CD133 degradation [Figs 3B, 4D] and thereby it facilitates anticancer therapeutic efficacy in HCC *in vivo* [Fig. 5].

Actually, we herein had planned an experiment to observe effects of combination therapy with oxytetracycline and existing anticancer therapies *in vivo* in HCC. However, many medications known to interact with oxytetracycline occurs various side effects after combination treatment with oxytetracycline. Representatively, there are actretin, cholera vaccine, etretinate, isotretinoin, lomitapide, methoxyflurane, mipomersen, tretinoin, vitamin A *etc*., which are should avoid combination treatment with oxytetracycline. To avoid certain type of side effects caused by combination therapy with oxytetracycline, we have just investigated anticancer efficacy of single treatment of oxytetracycline. Interestingly, treatment of oxytetracycline significantly attenuated tumor formation and CD133^+^ cell population in HCC-xenograft mice [Fig. 5].

In this paper, we have developed an *in vitro* co-culture system of HCC cells with hepatocytes to recapitulate tumor heterogeneity to identify CD133^+^ HCC-specific drugs in co-cultures of HCC cells with hepatocytes and have performed image-based phenotypic drug screening for the development of HCC therapeutics. This *in vitro* screening platform has the potential to amplification be widely adopted in drug discovery research due to its easy handling and efficiency. Based on the image-based phenotypic analysis, we promptly estimated elimination activity of CD133^+^ HCC and hepatotoxicity and elucidated functional roles of oxytetracycline on the preservation of CD133 stability in HCC.

Therefore, our results suggest that oxytetracycline might be new candidate approach to amplification of therapeutic efficacies in liver cancer and should significantly reduce the costs of cancer patient care, making treatment more accessible in the developing world.

## Electronic supplementary material

Supplementary Information
