# Supplementary material for: Oxytetracycline have the therapeutic efficiency in CD133+ HCC population through suppression CD133 expression by decreasing of protein stability of CD133
Source: Sci Rep. 2018 Oct 31;8:16100. doi: 10.1038/s41598-018-34301-1 (PMC6208387; doi:10.1038/s41598-018-34301-1)
Supplement: Supplementary file 1 — Supplementary Information [file 41598_2018_34301_MOESM1_ESM.pdf]

# Oxytetracycline have the therapeutic efficiency in CD133$^+$ HCC population through suppression CD133 expression by decreasing of protein stability of CD133

Yeonhwa Song[1], In-Ki Kim[2], Inhee Choi[3], Se-Hyuk Kim[1] and Haeng Ran Seo[1*]

## Author Affiliations:

[1] Cancer Biology Laboratory, Institut Pasteur Korea, 16, Daewangpangyo-ro 712 beon-gil, Bundang-gu, Seongnam-si, Gyeonggi-do, 13488, Korea, [2] Department of Convergence Medicine, University of Ulsan College of Medicine and Asan Institute for Life Sciences, ASAN Medical center, Olympic-ro 43-gil, Songpa-gu, Seoul, 05505, Korea. [3] Medicinal Chemistry, Institut Pasteur Korea, 16, Daewangpangyo-ro 712 beon-gil, Bundang-gu, Seongnam-si, Gyeonggi-do, 13488, Korea

* Corresponding author: Haeng Ran Seo, Cancer Biology Research Laboratory, Institut Pasteur Korea, 16, Daewangpangyo-ro 712 beon-gil, Bundang-gu, Seongnam-si, Gyeonggi-do, 13488 Republic of Korea; Tel: +82-31-8018-8300; E-mail: shr1261@ip-korea.org

## Supplementary Figure Legends

**Supplementary Figure 1.** Images of AFP and RFP in HCC-mixed culture system with hepatocellular carcinoma (Huh7.5-RFP) and immortalized normal hepatocyte (Fa2N-4).

**Supplementary Figure 2**. Degradation of CD133 protein by treatment of 100μM oxytetracycline at indicated time in Huh7.

**Supplementary Figure 3.** Expression of cancer stem cells-related markers in Huh7 and Hep3B cells after treatment of oxytetracycline with indicated concentration.

**Supplementary Figure 4.** Measurement of mRNA expression of stem cell-related markers by real-time polymerase chain reaction **(A)** and reverse transcription-polymerase chain reaction **(B)** in Huh7 and He3B cells after treatment of oxytetracycline with indicated concentration

**Supplementary Figure 5** Stability of CD133 protein by treating with doxycycline and tetracycline with indicated concentration in Huh7 cells **(A)** and Hep3B cells **(B).**

**Supplementary Figure 6.** Body weight of HCC-xenograft mice models

# Supplementary Figure 1

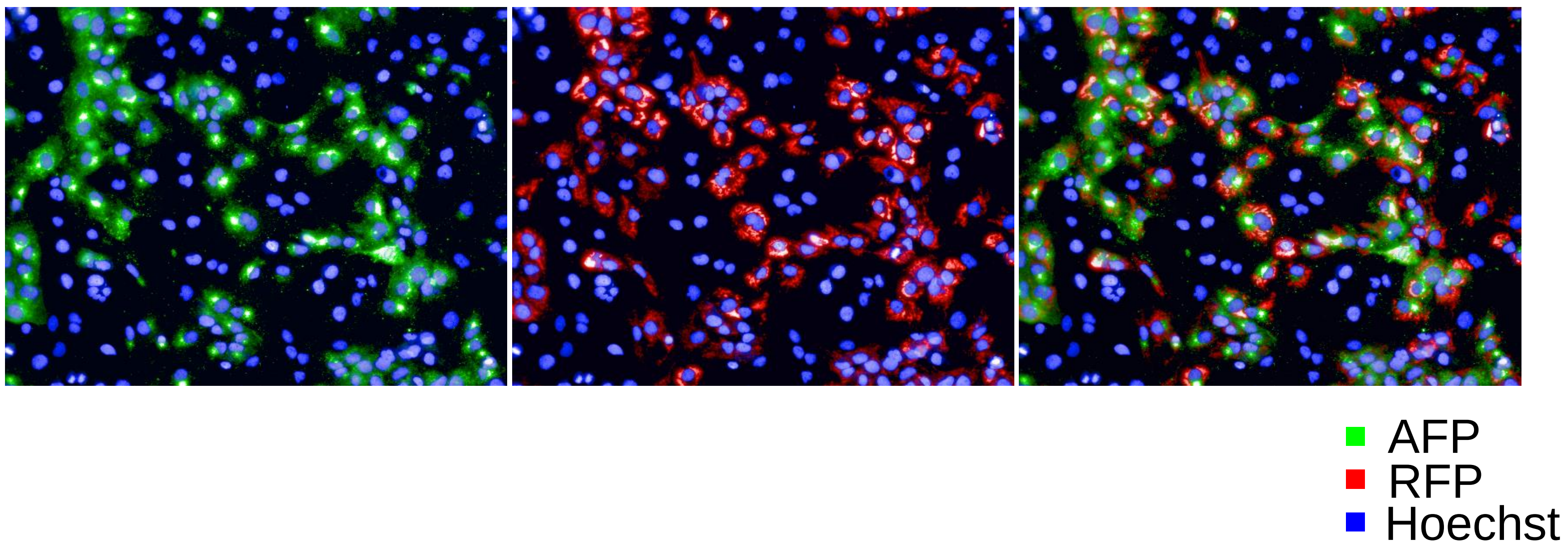

# Supplementary Figure 2

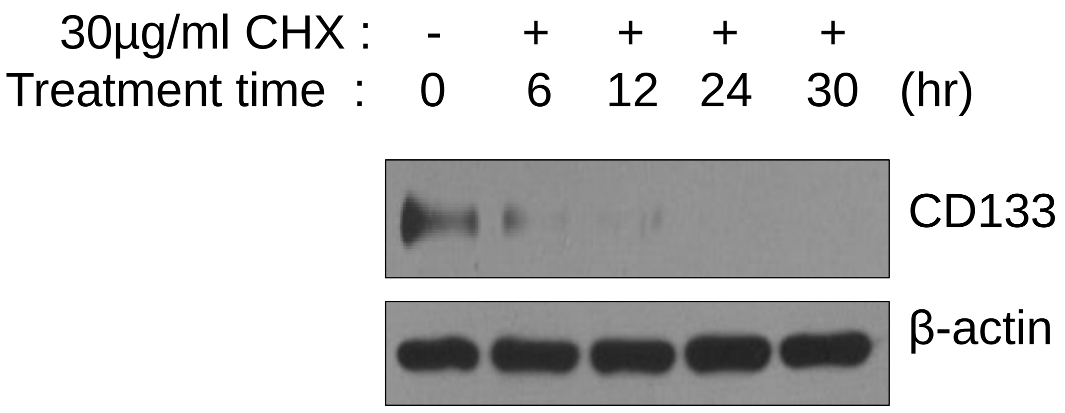

# Supplementary Figure 3

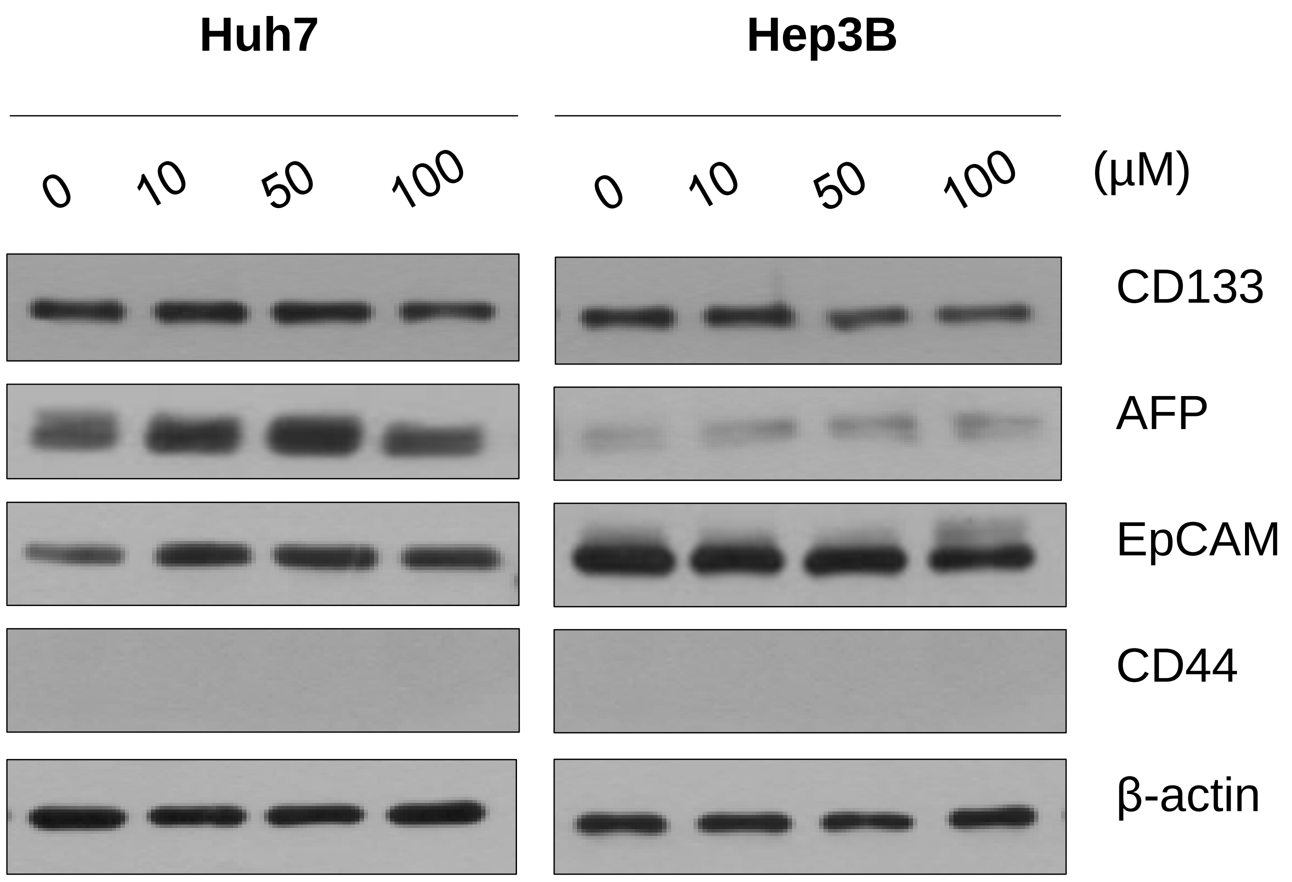

# Supplementary Figure 4

A.

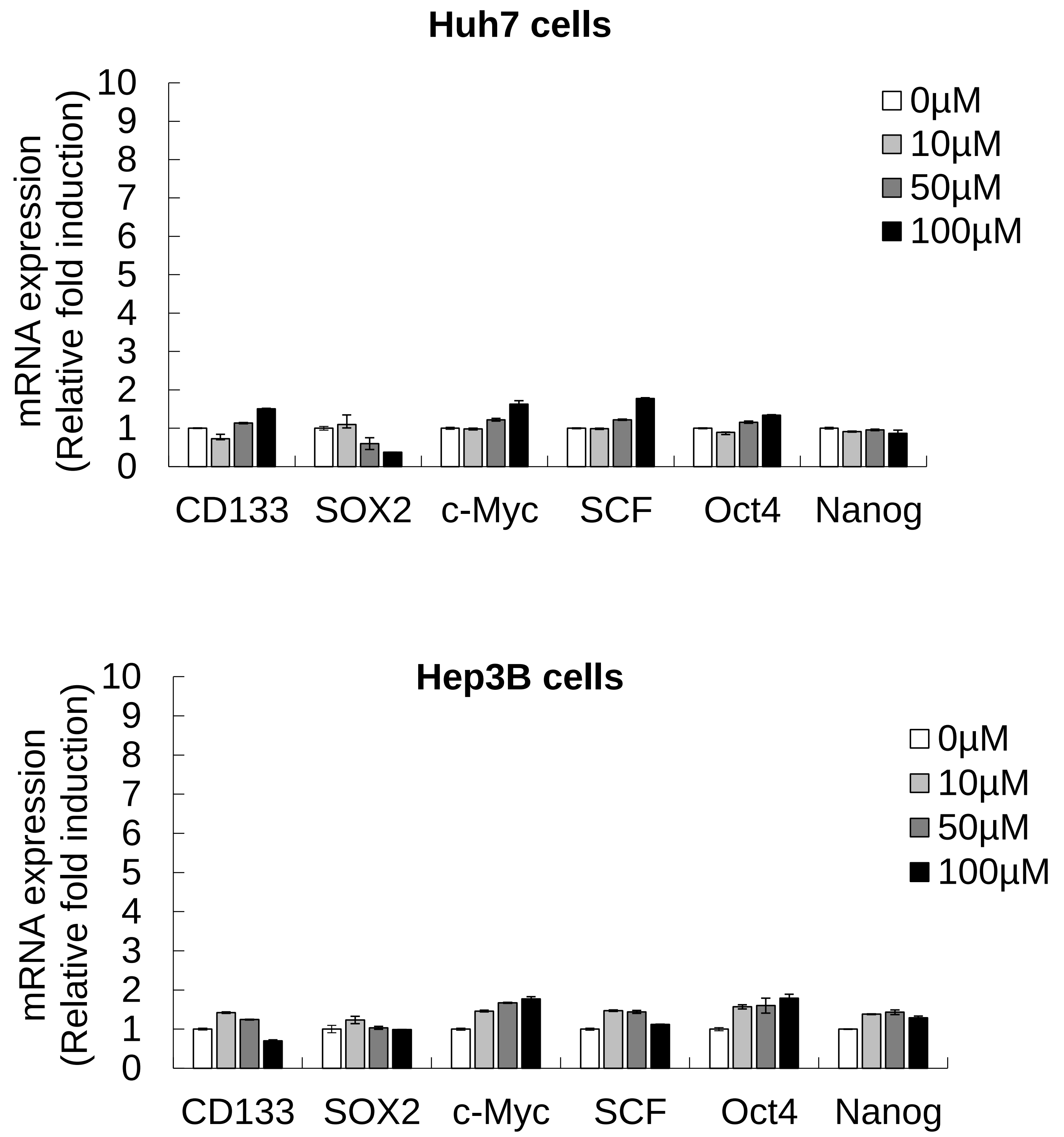


B.

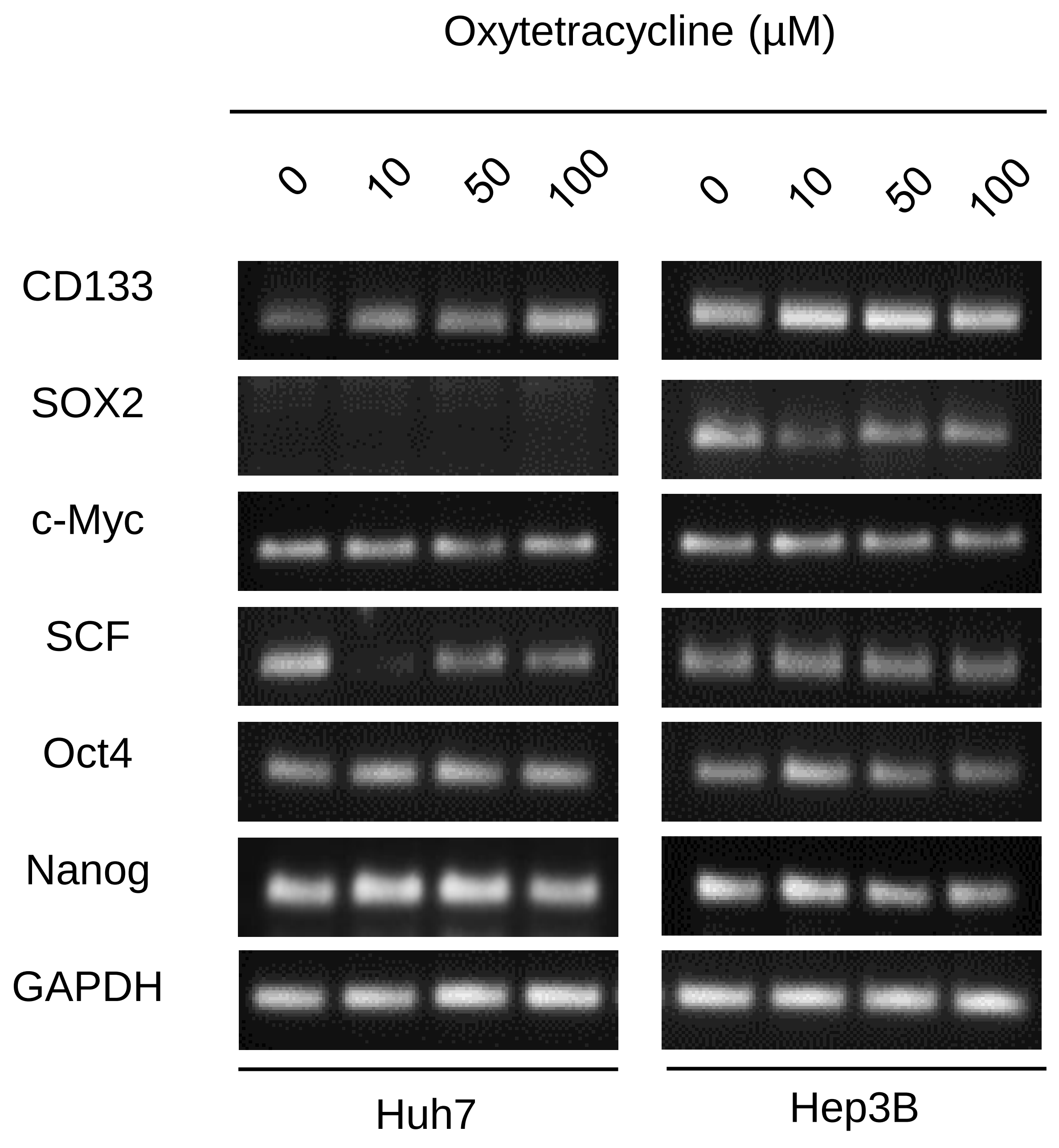

# Supplementary Figure 5

## A.

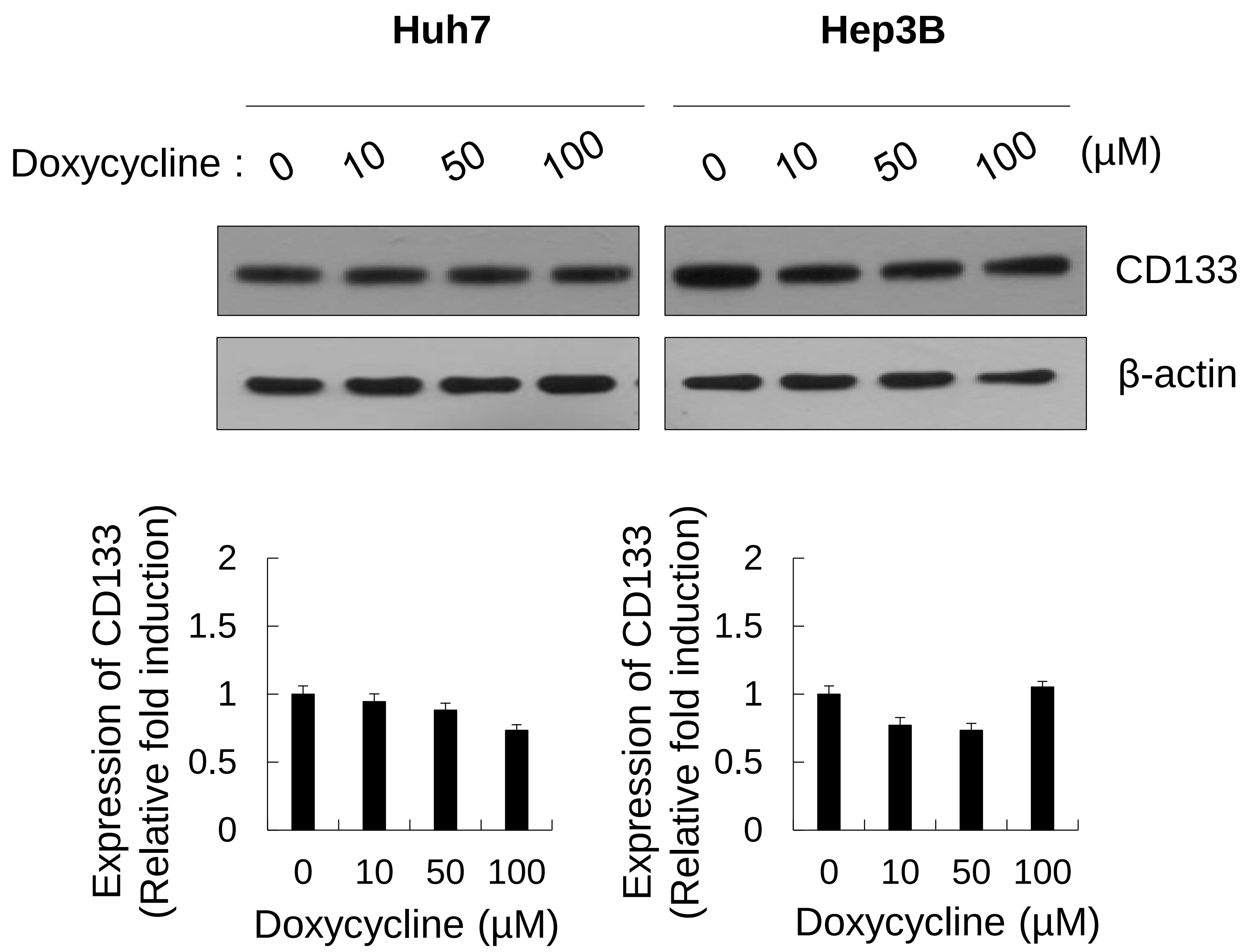


## B.

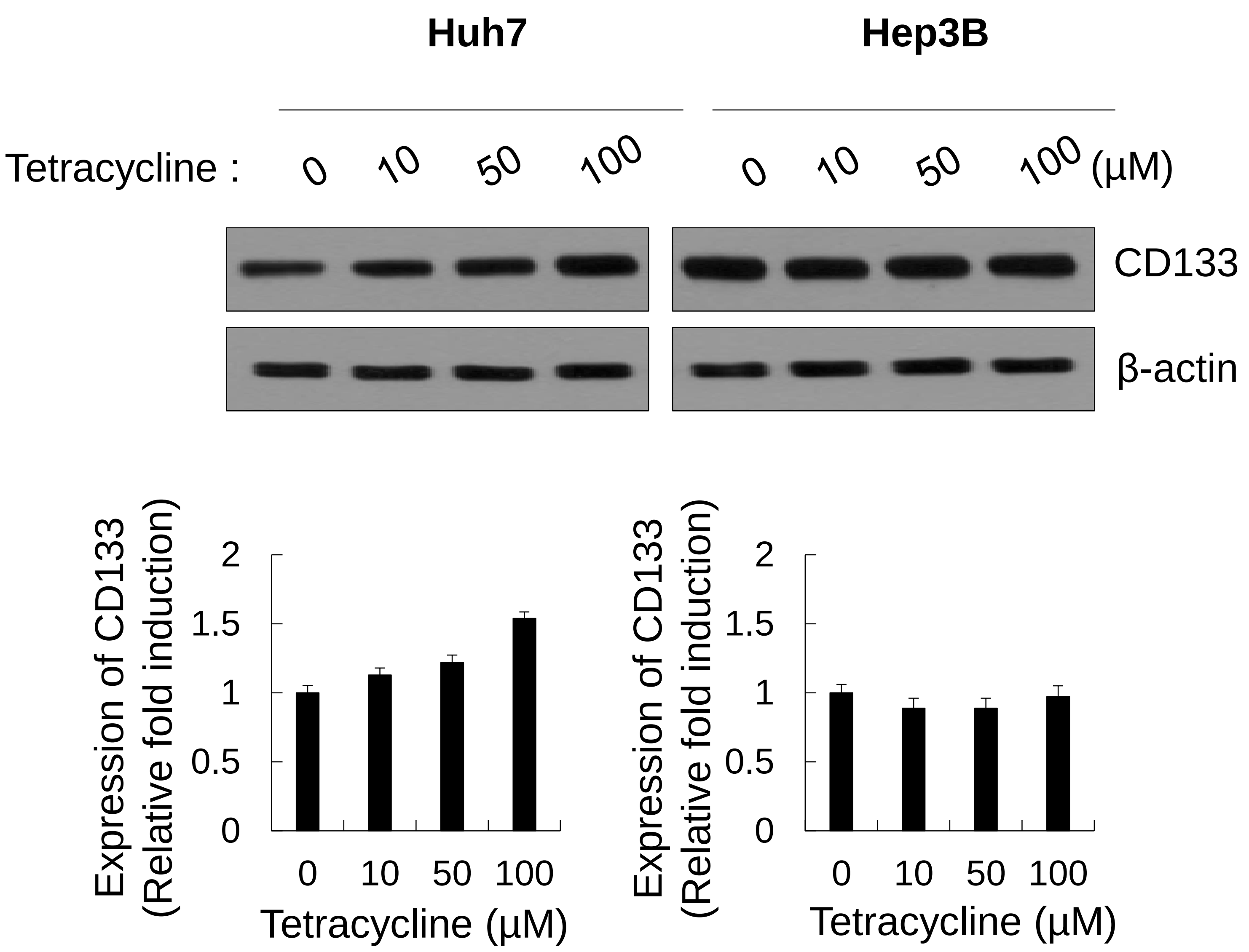

# Supplementary Figure 6

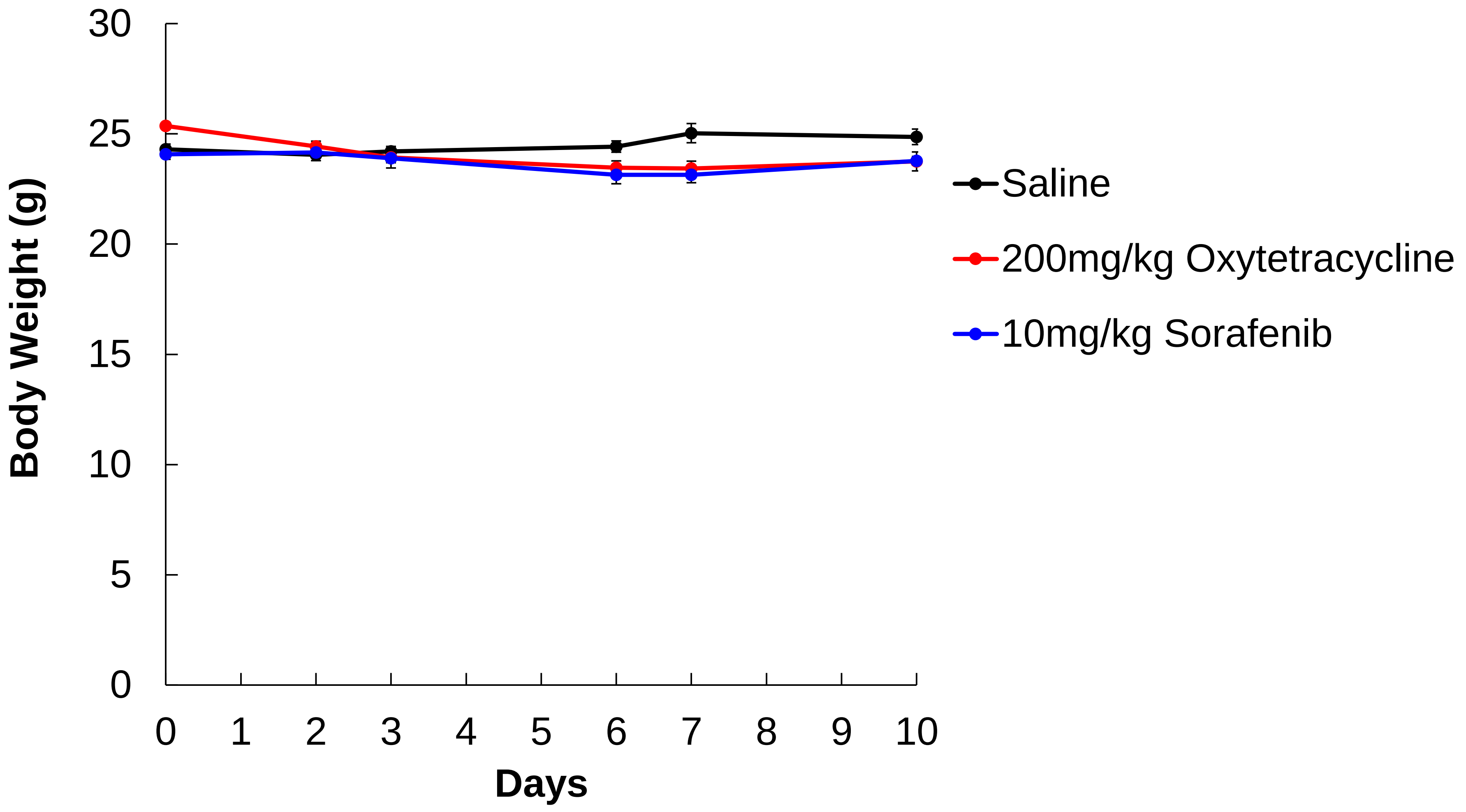

2016. 11. 4. (THU) - CD133 screening hit Validation. Figure 3B.

Cell: Hm7.5

Drug: β-chloro-L-alanine, Oxytetracycline, Fusidic acid, LY-294,002.

Drug Concentration: 0, 10uM, 50uM

Drug treatment: 48hr

anti-CD133
(130KDa)

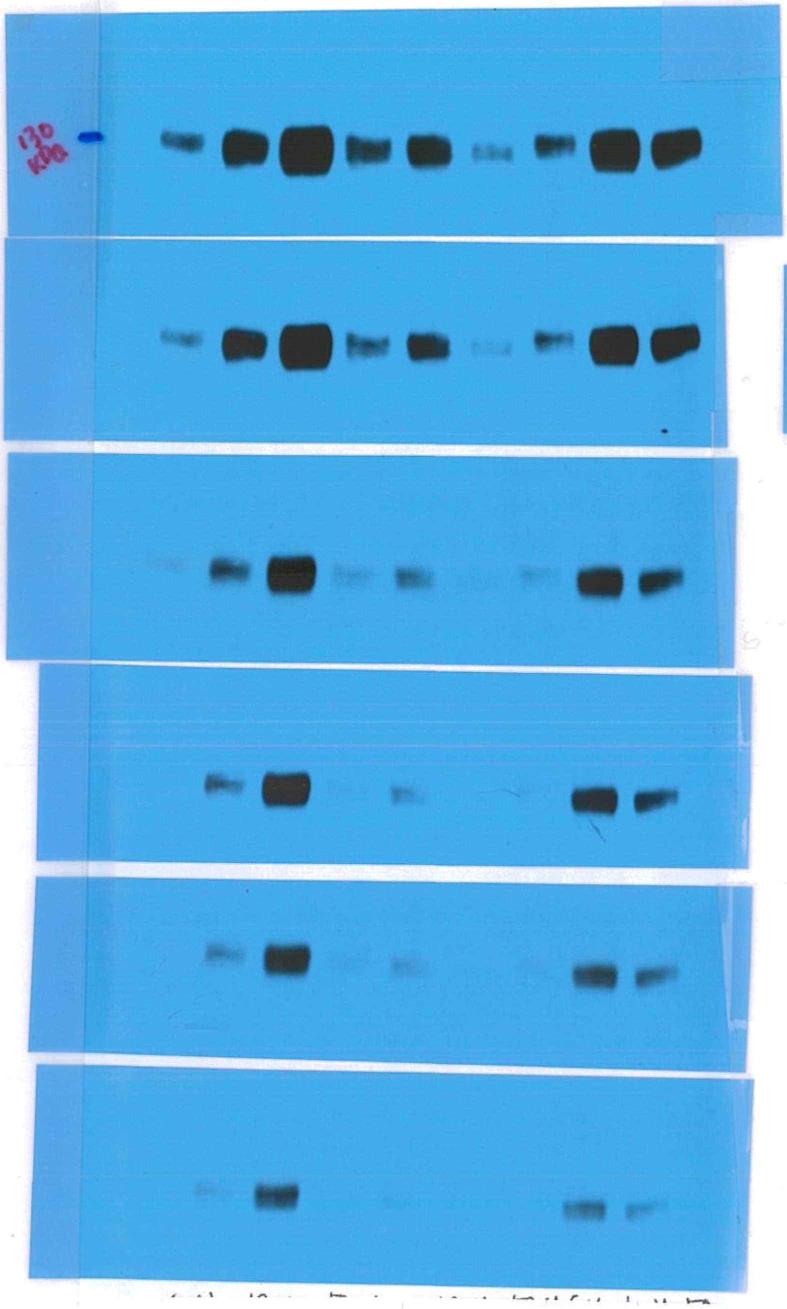


0 10 50 0 10 50 0 10 50 (uM)

β-chloro-L-alanine | Oxytetra-cycline | Fusidic Acid

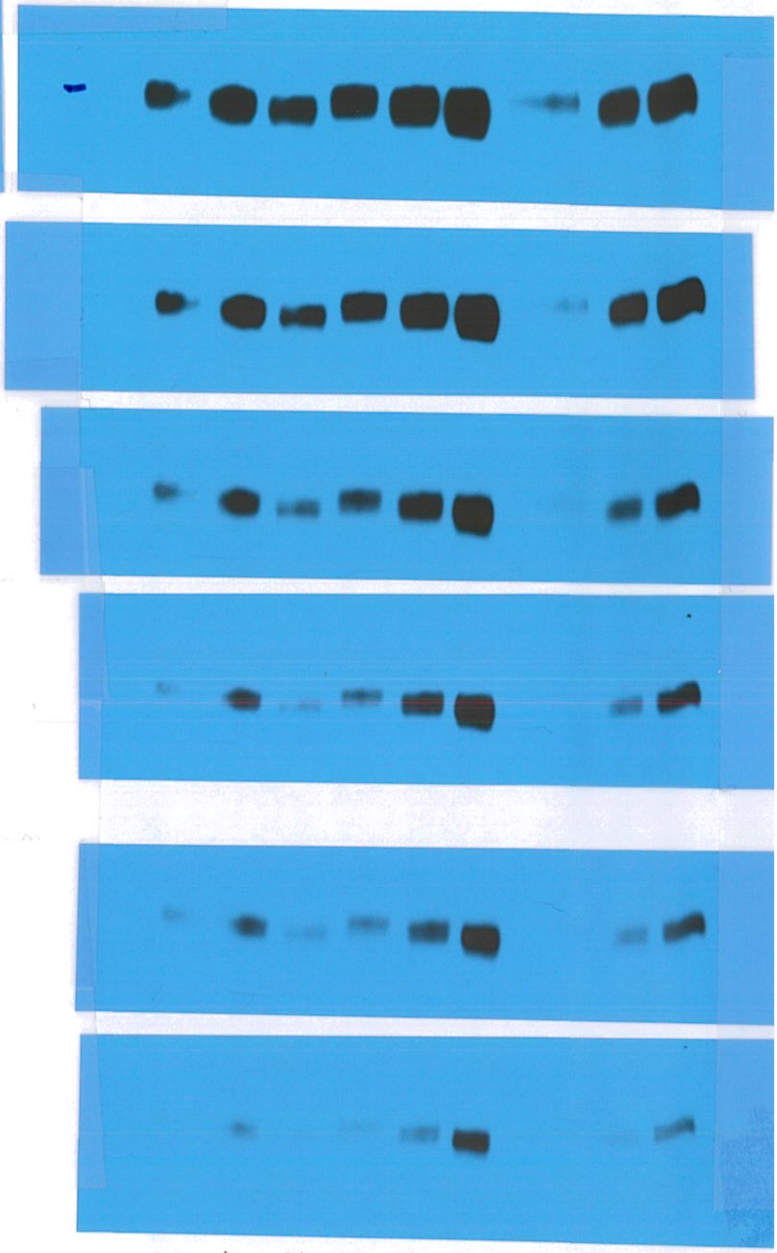

0 10 50 0 1 5 0 1 5 (uM)

LY-294,002 | Sorafenib | Regorafenib.

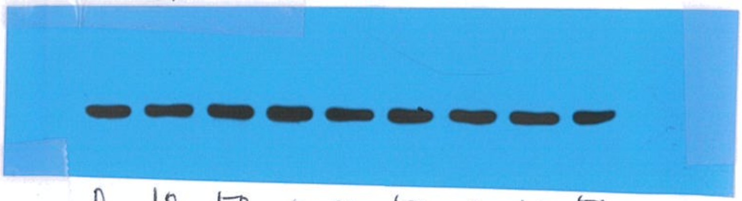

β-actin

0 10 50 0 10 50 0 10 50

β-chloro-L-alanine | LY-294002 | Oxytetra

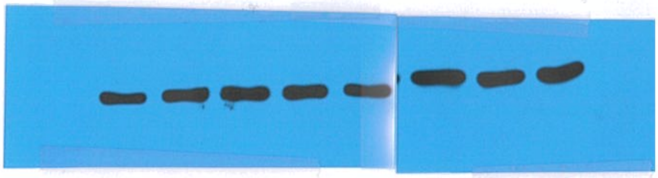

0 10 50 X 0 10 50

Fusidic acid | Sorafenib

β-a

2017.06.19. CD133 Stability

Figure 4D.E

• Cell: Huh7. Hep3B

• Compound: Oxytetracycline (0.10.50.100μM)

CHX (30μg/mL)

• Time: 48hr.

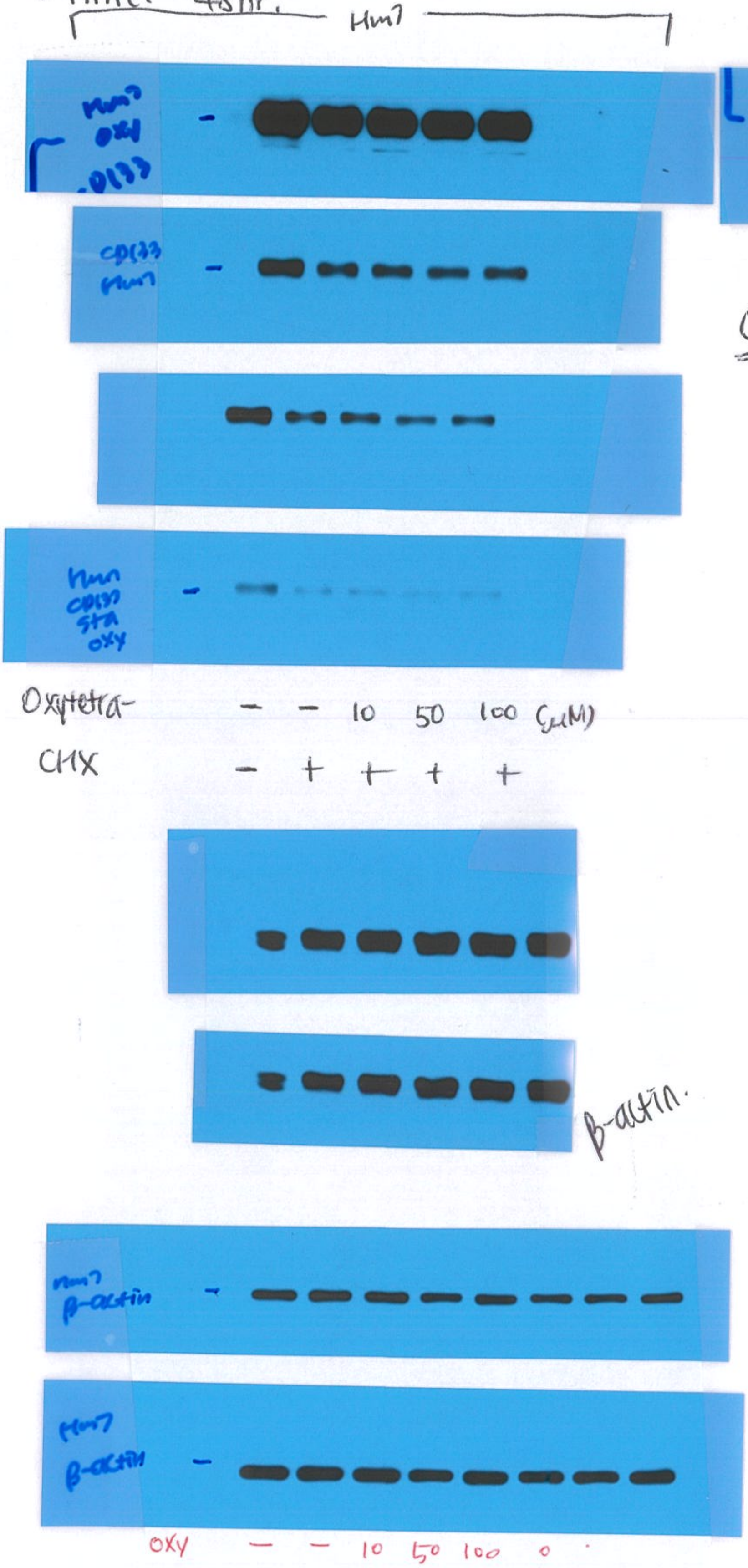


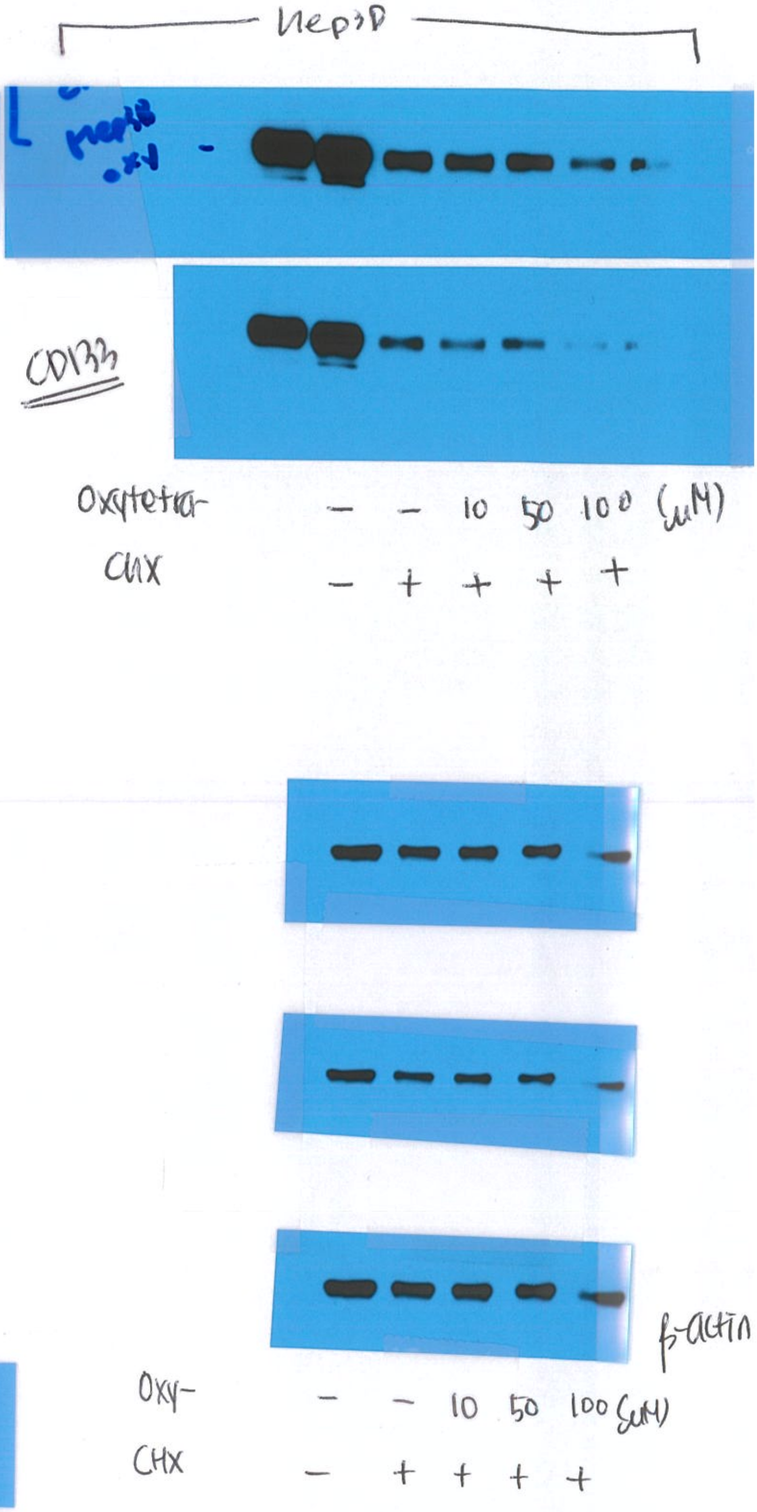

2018.02.03 CD133 Stability.

Supple Figure 2

Cell: Huh7. Hep3B

Condition: CHX 30ng/mL with/without
Oxytetracycline 100nM (0.6.12.24.30hr)

Huh7

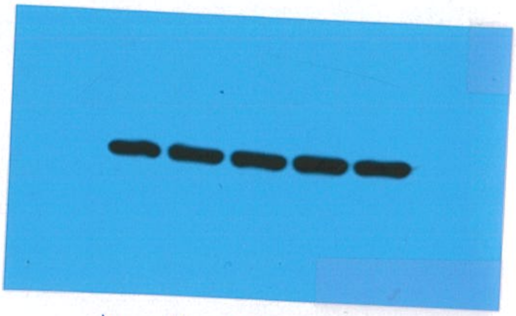

Hep3B

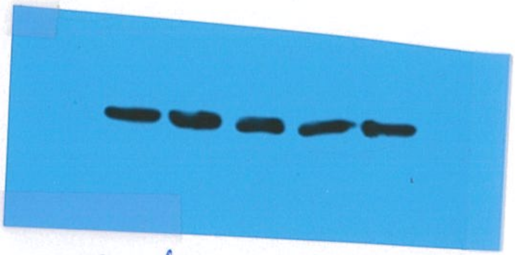

β-actin

Oxytetra 0hr 6hr 12hr 24hr 30hr
CHX + + + + +

0hr 6hr 12hr 24hr 30hr
+ + + + +

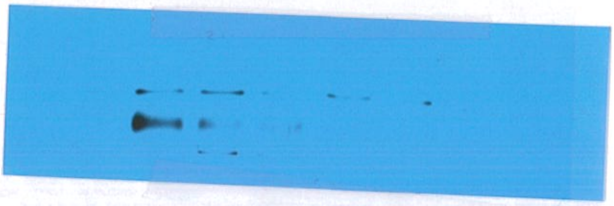

CD133.

2017.06.26 · Oxytetracycline – CSC marker. (Huh7. Hep3B)

Cells : Huh7. Hep3B

Con. : 0. 10. 50. 100uM 48hr.

| | 7 | 3B |
|---|---|---|
| AFP | +++ | + |
| EpCAM | + | +++ |
| CD90 | + | – |
| CD24 | ? | ? |
| CD44 | – | – |

Sopple Figure

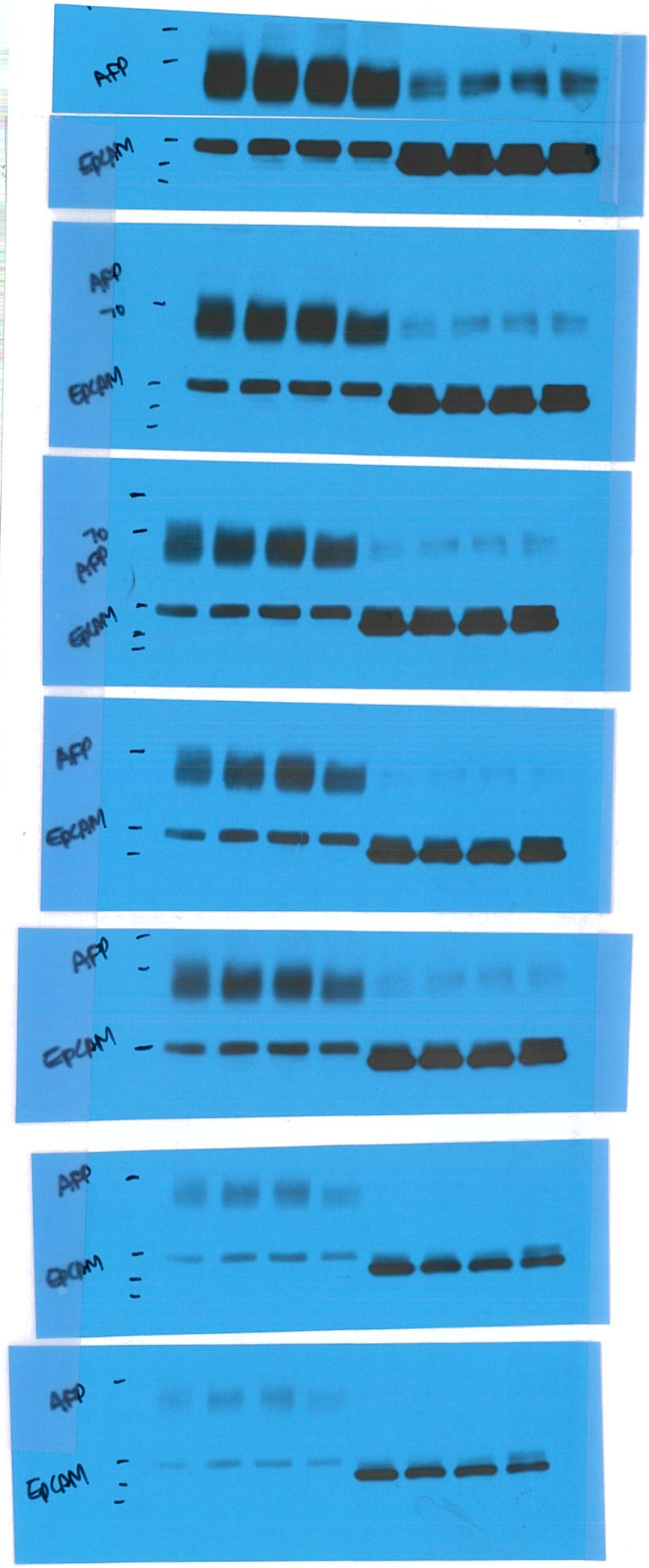


OXY: 0 10 50 100 0 10 50 100 (uM)

Huh7 Hep3B.

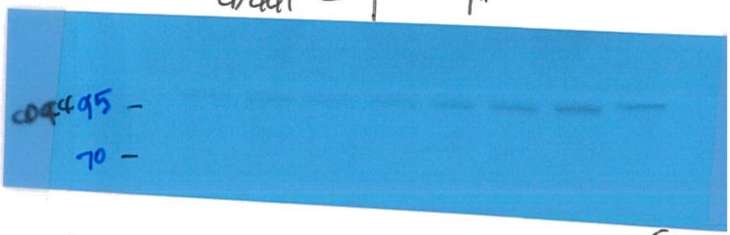


CD44

CD90X.

2017.08.09. Doxycycline. Tetracycline. – Huh7. Hep3B.

Supple Figure 5.

1. Huh7 Doxy 0. 10. 50. 100 uM / Hep3B Doxy 0. 10. 50. 100 uM

2. Huh7 Tetra– " / " Tetra "

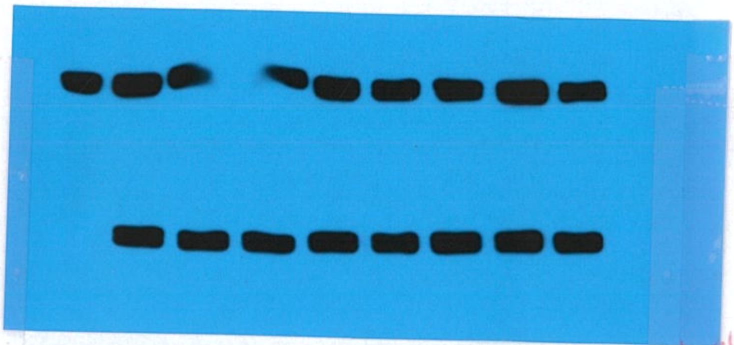

β-actin

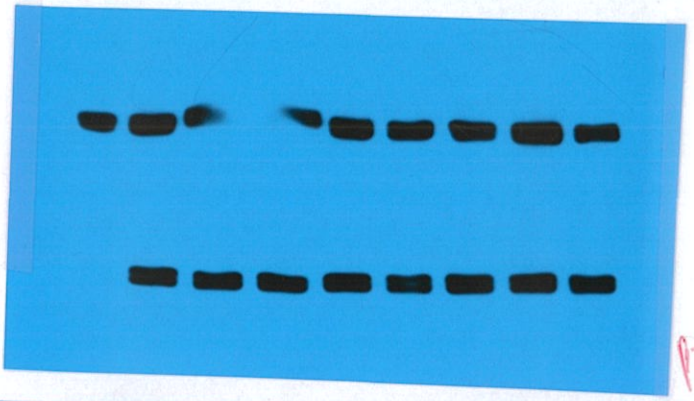

β-actin

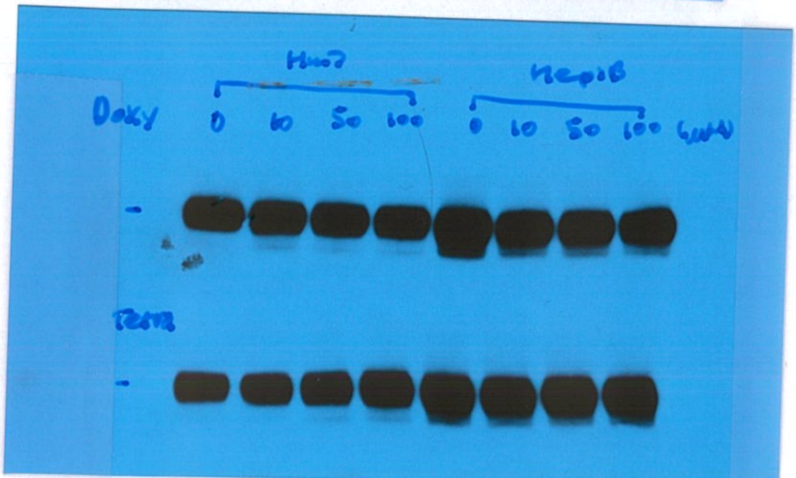


CD133

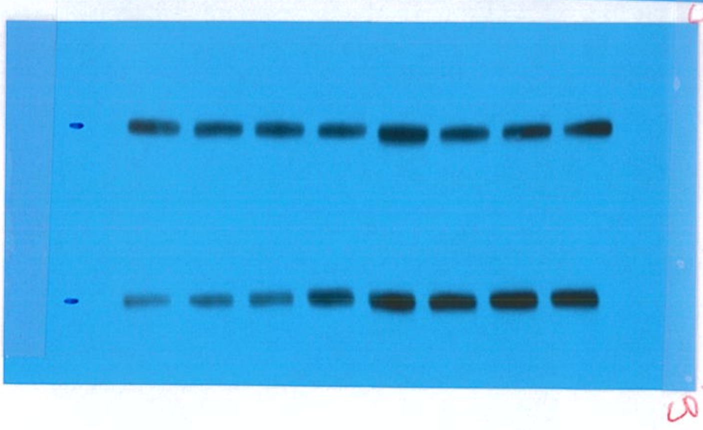

CD133

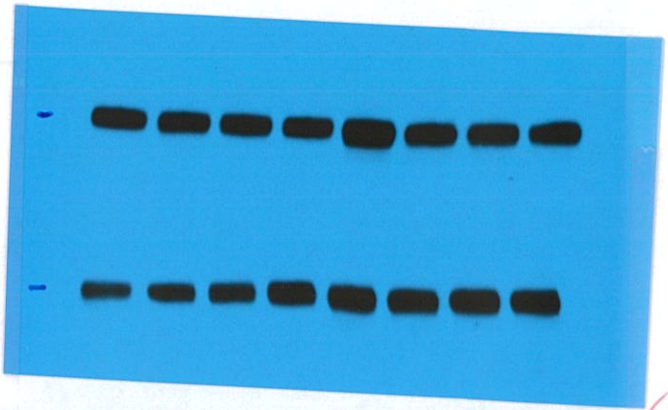

C

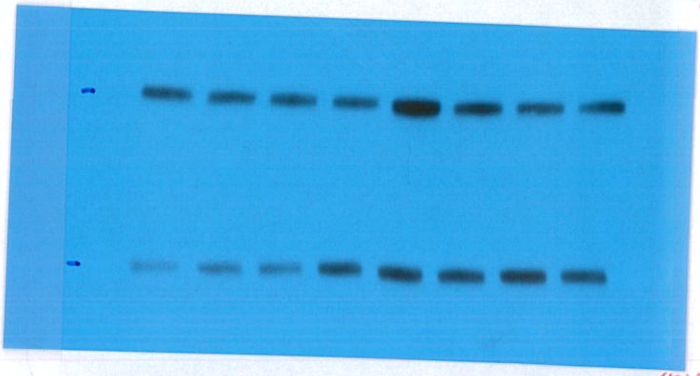

CD133

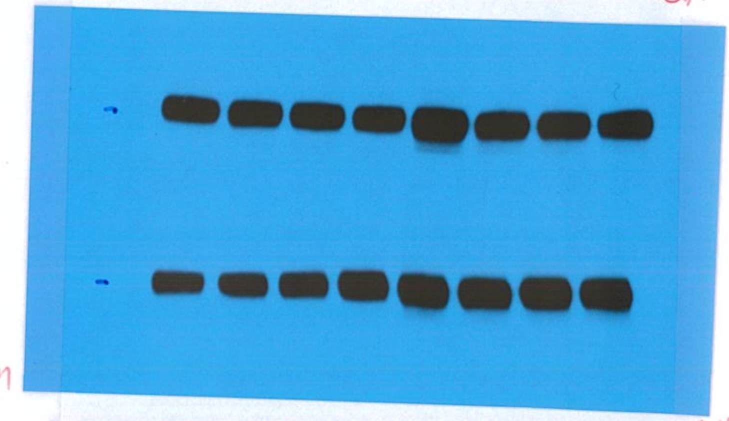

CD13

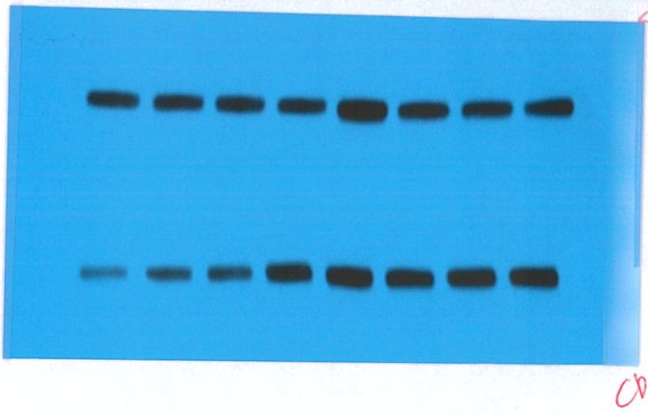

CD133
